# PFKFB2‐Driven Glycolysis Promotes Dendritic Cell Maturation and Exacerbates Acute Lung Injury

**DOI:** 10.1002/advs.202502428

**Published:** 2025-07-30

**Authors:** Ding Yuan, Fang Yang, Linlin Hou, Yan Zhang, Xin Pang, Yuqi Du, Hongyi Yan, Huanzhou Zhu, Yue Cheng, Yue Wu, Pinpin Jiang, Mengnan Guo, Mengying Zhang, Jinjie Guo, Huihui Hao, Yong Jiang, Yi Li, Yanxia Gao

**Affiliations:** ^1^ Emergency Department Medical Key Laboratory of Poisoning Diseases of Henan Province The First Affiliated Hospital of Zhengzhou University Zhengzhou Henan 450052 China; ^2^ School of Pharmacy Henan University of Traditional Chinese Medicine Zhengzhou 450046 China; ^3^ Henan International Joint Laboratory of Infection and Immunity The First Affiliated Hospital of Zhengzhou University Zhengzhou 450052 China; ^4^ Henan Key Laboratory of Critical Care Medicine Emergency Department The First Affiliated Hospital of Zhengzhou University Zhengzhou 450052 China; ^5^ Institute of Infection and Immunity Henan Academy of Innovations in Medical Science Zhengzhou 451163 China; ^6^ Emergency Department State Key Laboratory of Complex Severe and Rare Diseases Peking Union Medical College Hospital Chinese Academy of Medical Science and Peking Union Medical College Beijing 100730 China

**Keywords:** acute lung injury, dendritic cells, glycolysis, HIF‐1α, PFKFB2

## Abstract

Acute lung injury (ALI) is a life‐threatening condition with excessive immune activation and dysregulated inflammation. Dendritic cells (DCs) play a pivotal role in immune regulation; however, their exact contribution to ALI pathogenesis remains unclear. This study demonstrates that the upregulation of the glycolytic regulator 6‐phosphofructo‐2‐kinase/fructose‐2,6‐bisphosphatase 2 (PFKFB2) by hypoxia‐inducible factor‐1α (HIF‐1α) enhances glycolysis, drives DC maturation, and exacerbates inflammation, contributing to the pathogenesis of ALI. The findings reveal that HIF‐1α directly binds to the PFKFB2 promoter and drives its transcription, leading to increased glycolysis, accelerated DC maturation, and amplified immune activation. In paraquat (PQ)‐ALI and lipopolysaccharide (LPS)‐ALI mouse models, DC‐specific PFKFB2 knockout and DC‐targeted delivery of HIF‐1α inhibitor‐loaded nanoparticles each significantly suppressed DC maturation and alleviated ALI severity. Analyses of lung tissues from patients with PQ poisoning, secondary bacterial pneumonia (2°BP), and Coronavirus Disease 2019 (COVID‐19), as well as from normal controls, confirmed these findings, showing increased PFKFB2 expression and DC maturation during ALI. These findings highlight the HIF‐1α–PFKFB2 signaling pathway as a critical regulator of glycolysis‐driven DC maturation and immune activation, offering novel insights into immunometabolic regulation and a promising therapeutic target for ALI.

## Graphical abstract

Paraquat (PQ) or lipopolysaccharide (LPS) exposure activates the HIF‐1α–PFKFB2 pathway, driving glycolysis in dendritic cells (DCs). This metabolic shift enhances DC maturation and immune activation, exacerbating acute lung injury (ALI). This study demonstrates that PFKFB2‐driven glycolytic reprogramming in DCs plays a critical role in the pathogenesis of ALI, highlighting it as a potential therapeutic target.

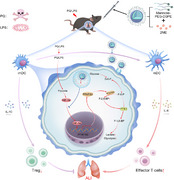

## 1 Introduction

Acute lung injury (ALI) is a severe clinical condition with an incidence ranging from 10.1 to 86.2 per 1 00,000 person‐years and a mortality rate reaching up to 30–40%.^[^ ^1^ ^]^ The condition arises from diverse etiologies, including sepsis, bacterial pneumonia, multiple traumas, aspiration pneumonia, and chemical lung injury.^[^ ^2^ ^]^ Despite these varied causes, ALI is unified by common pathological features, such as dysregulated systemic inflammation, oxidative stress, increased endothelial and epithelial permeability, and diffuse alveolar damage. These pathological changes lead to alveolar edema, fibrosis, necrosis, and the deposition of proteinaceous exudates, which collectively impair respiratory function.^[^ ^3^, ^4^ ^]^ The pathophysiological mechanism underlying these changes is driven by an imbalance in the inflammatory response, wherein an excessive immune reaction triggers a deleterious inflammatory cascade. Given the pivotal role of the innate immune response as the first line of defense in ALI, its regulation is crucial for mitigating excessive inflammation.^[^ ^5^ ^]^ Consequently, modulating immune responses has emerged as a promising therapeutic strategy for ALI.

Dendritic cells (DCs), renowned for their potent antigen‐presenting capacity and highly sensitive sentinel network, play a central role in initiating and regulating immune responses during pulmonary infections and inflammation.^[^ ^5^ ^]^ Mature dendritic cells (mDCs) are particularly effective in inducing the proliferation and activation of naive T cells, thereby amplifying immune responses.^[^ ^6^, ^7^ ^]^ In the ALI animal model, the number of mDCs in lung tissue significantly increased, suggesting that the maturation of lung DCs contributes to acute pulmonary inflammation and pathological injury. Inhibition of DCs recruitment and maturation can significantly improve lipopolysaccharide (LPS) induced ALI in mice.^[^ ^8^, ^9^ ^]^ These findings underscore the potential of targeting DC maturation as a therapeutic approach to modulate immune responses in ALI. Emerging evidence highlights the critical role of cellular metabolism in DC maturation, particularly the metabolic shift from oxidative phosphorylation to glycolysis. This metabolic reprogramming is essential for meeting the energy and biosynthetic demands of DC maturation.^[^ ^10^ ^]^ Inhibition of glycolysis has been shown to impair DCs migration and maturation, leading to reduced expression of major histocompatibility complex II (MHC‐II), costimulatory molecules (CD86/CD40), and pro‐inflammatory cytokines.^[^ ^11^ ^]^

Phosphofructo‐2‐kinase/fructose‐2,6‐bisphosphatase 2 (PFKFB2) is a key regulatory enzyme in glycolysis. It enhances the synthesis of fructose‐2,6‐bisphosphate (F2,6BP), thereby activating the rate‐limiting glycolytic enzyme phosphofructokinase‐1 (PFK1) and promoting glycolytic metabolism.^[^ ^12^ ^]^ Previous studies have demonstrated that PFKFB2‐mediated glycolysis plays an important role in the pathogenesis of various diseases, including glioblastoma,^[^ ^13^ ^]^ cardiovascular disorders,^[^ ^14^ ^]^ and pancreatitis.^[^ ^15^ ^]^ In the context of immune regulation, PFKFB2‐driven glycolysis has been shown to enhance neutrophil inflammatory responses,^[^ ^16^ ^]^ promote M1 macrophage polarization,^[^ ^17^ ^]^ and sustain lactate‐driven efferocytosis in macrophages.^[^ ^18^ ^]^ These findings indicate that PFKFB2‐mediated glycolysis contributes to the regulation of immune cell functions; however, its specific role in DCs remains largely undefined. A previous study reported that hypoxia can upregulate PFKFB2 transcription,^[^ ^19^ ^]^ possibly through the activation of hypoxia‐inducible factor‐1α (HIF‐1α).^[^ ^13^ ^]^ Notably, previous studies have shown that both paraquat (PQ) and LPS‐induced ALI are associated with significant upregulation of HIF‐1α,^[^ ^20^, ^21^, ^22^ ^]^ and HIF‐1α has been implicated in the regulation of LPS‐induced DC maturation and function.^[^ ^23^ ^]^ Collectively, these findings suggest that the HIF‐1α‐PFKFB2 axis may contribute to DC maturation via glycolytic reprogramming during PQ/LPS‐induced ALI, thereby exacerbating inflammation and lung injury.

In this study, we demonstrated the role of PFKFB2 in regulating the maturation of pulmonary DCs during ALI. Specifically, we investigated the transcriptional regulation of PFKFB2 by HIF‐1α and explored the therapeutic potential of targeting this pathway. By employing DC‐specific conditional knockout of PFKFB2 and selective inhibition of HIF‐1α in DCs, we assessed the impact of disrupting the HIF‐1α‐PFKFB2 axis on lung injury and inflammation in both PQ/LPS‐induced ALI mouse models, and validated these findings in lung tissue samples from ALI patients.

## 2 Results

### 2.1 Time‐Dependent Effects of PQ on Lung Injury and DC Maturation

**Figure 1:**
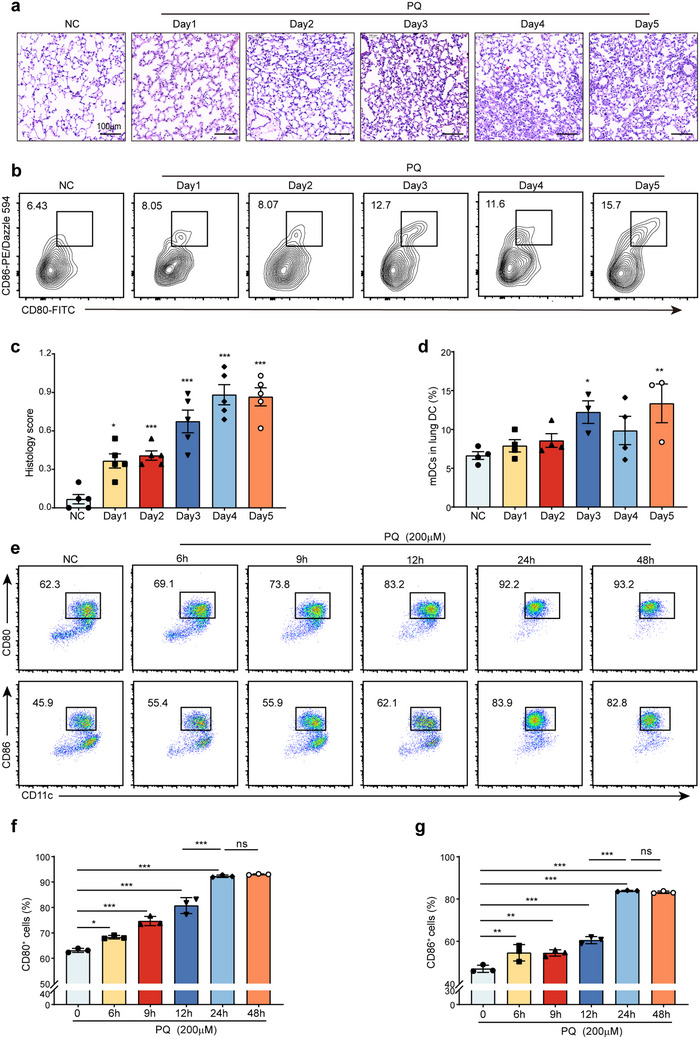
Correlation between PQ‐ALI and DC maturation. a,c) H&E staining of mouse lung tissue (*n* = 5 mice/group). b,d) Flow cytometry analysis of the proportion of mDCs in mouse lung tissue, with CD80^+^CD86^+^ cells representing mDCs (*n* = 3–4 mice/group). e‐g) Flow cytometry to assess the maturation proportion of BMDCs exposed to 200 µm PQ for different durations (0, 6, 9, 12, 24, 48 h) (*n* = 3 wells/group). *^ns^p >* 0.05, ^*^p < 0.05, ^*^ ^*^p < 0.01, ^*^ ^**^p < 0.001. All values are means ± SD, and significance was determined by one‐way analysis of variance (ANOVA) with Fisher's least significant difference (LSD) post hoc analysis.

The administration of PQ‐induced significant lung injury in a time‐dependent manner, as evidenced by histological analysis (**Figure** **1**a,c). Flow cytometry was employed to evaluate the expression of surface markers on lung DCs at various time points following PQ exposure. A time‐dependent increase in the proportion of lung mDC was observed, which correlated with the pathological scores of lung injury (Figure 1b,d).

To further investigate the impact of PQ on DC maturation, bone marrow‐derived dendritic cells (BMDCs) were cultured in vitro and exposed to PQ at concentrations ranging from 1 to 1,000 µm. PQ at 200 µm preserved approximately 90% cell viability while inducing the highest level of DC maturation, with viability decreasing dose‐dependently above this concentration (Figure S1e–i, Supporting Information). Time‐course experiments revealed that treatment with 200 µm PQ for durations of 6, 9, 12, 24, and 48 h resulted in a progressive increase in maturation rates, reaching a plateau at 24 h with no further significant changes observed at 48 h (Figure 1e–g). Therefore, this study selected a 200 µm PQ treatment of BMDCs for 24 h as the optimal condition to explore the effects of PQ on DC maturation. These conditions ensured both cell viability and a robust maturation response, providing a reliable model for subsequent mechanistic investigations.

### 2.2 PQ‐Induced Maturation of BMDCs is Dependent on Enhanced Glycolysis

**Figure 2:**
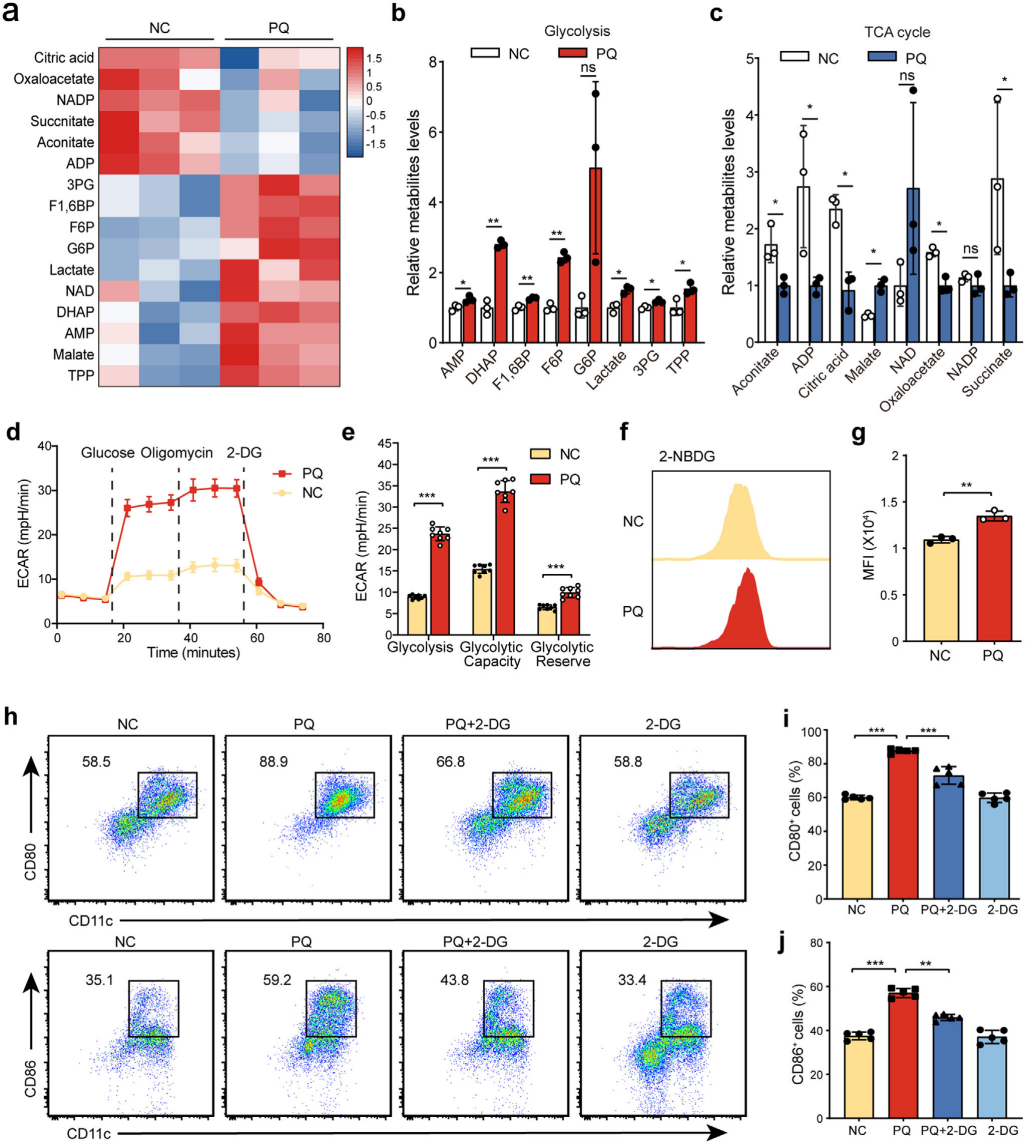
Glycolytic reprogramming and its essential role in PQ‐induced BMDC maturation. a) Heatmap of glycolytic and TCA cycle intermediates in BMDCs from NC and PQ‐treated groups. b,c) Quantification of key metabolites in glycolysis and the TCA cycle (*n* = 3 samples /group). d,e) Glycolytic stress test to measure the ECAR of BMDCs (*n* = 8 wells/group). f,g) Flow cytometry analysis of glucose uptake using the fluorescent glucose analog 2‐NBDG. h‐j) Flow cytometry to assess the maturation proportion of BMDCs treated with 20 µm 2‐DG and 200 µm PQ (*n* = 5 wells/group). *^ns^p* > 0.05, ^*^p < 0.05, ^**^p  < 0.01, ^***^p  < 0.001. Horizontal lines indicate the means ± SD, and significance was determined by two‐tailed Student's *t*‐test (a–c, f–g), two‐tailed Mann–Whitney U test (d–e), or one‐way ANOVA with Fisher's LSD post hoc analysis (h–j). n.s., not significant (*p*  >  0.05).

To elucidate the impact of PQ on the glycolysis of BMDCs, LC‐MS was employed to analyze metabolic products. Metabolic profiling revealed a significant shift in the metabolic pathway of PQ‐treated BMDCs, characterized by an increase in intermediate metabolites of glycolysis and a concomitant decrease in intermediates of the tricarboxylic acid (TCA) cycle compared to the non‐treated control (NC) group (**Figure** **2**a–c). To further confirm the glycolytic shift, a glycolytic stress test was conducted to measure the extracellular acidification rate (ECAR), an indicator of glycolytic activity. PQ‐treated BMDCs exhibited significantly enhanced glycolysis, glycolytic capacity, and glycolytic reserve compared to the NC group (Figure 2d,e). This metabolic reprogramming aligns with the observed increase in glycolytic intermediates, reinforcing the notion that PQ promotes a glycolytic phenotype in BMDCs.

Previous studies have reported that enhanced glycolysis is often accompanied by increased glucose uptake.^[^ ^24^, ^25^ ^]^ To validate this, the fluorescent glucose analog 2‐NBDG was used to measure glucose uptake in BMDCs. Flow cytometry analysis revealed that PQ treatment significantly increased glucose uptake in BMDCs compared to the NC group (Figure 2f,g). This observation aligns with the enhanced glycolytic activity detected in PQ‐treated BMDCs, as increased glucose uptake is a hallmark of glycolytic reprogramming. The above results can explain the phenomenon of PQ driving the metabolism of BMDCs toward glycolysis. Given the concurrent enhancement of BMDC maturation and glycolysis following PQ exposure, the study sought to determine whether glycolysis was essential for PQ‐induced BMDC maturation. To address this, BMDCs were treated with the glycolysis inhibitor 2‐deoxy‐D‐glucose (2‐DG) in the presence of PQ, and we found that 2‐DG significantly reduced the maturation of BMDCs induced by PQ (Figure 2h–j). Consistently, 2‐DG treatment markedly decreased the levels of TNF‐α, IL‐6, and IL‐1β, while increasing IL‐10 expression in PQ‐stimulated BMDCs (Figure S3a–d, Supporting Information). This finding establishes glycolysis as a critical metabolic pathway required for the maturation of BMDCs in response to PQ exposure.

### 2.3 PQ Regulates DC Maturation via PFKFB2‐Mediated Glycolysis

**Figure 3:**
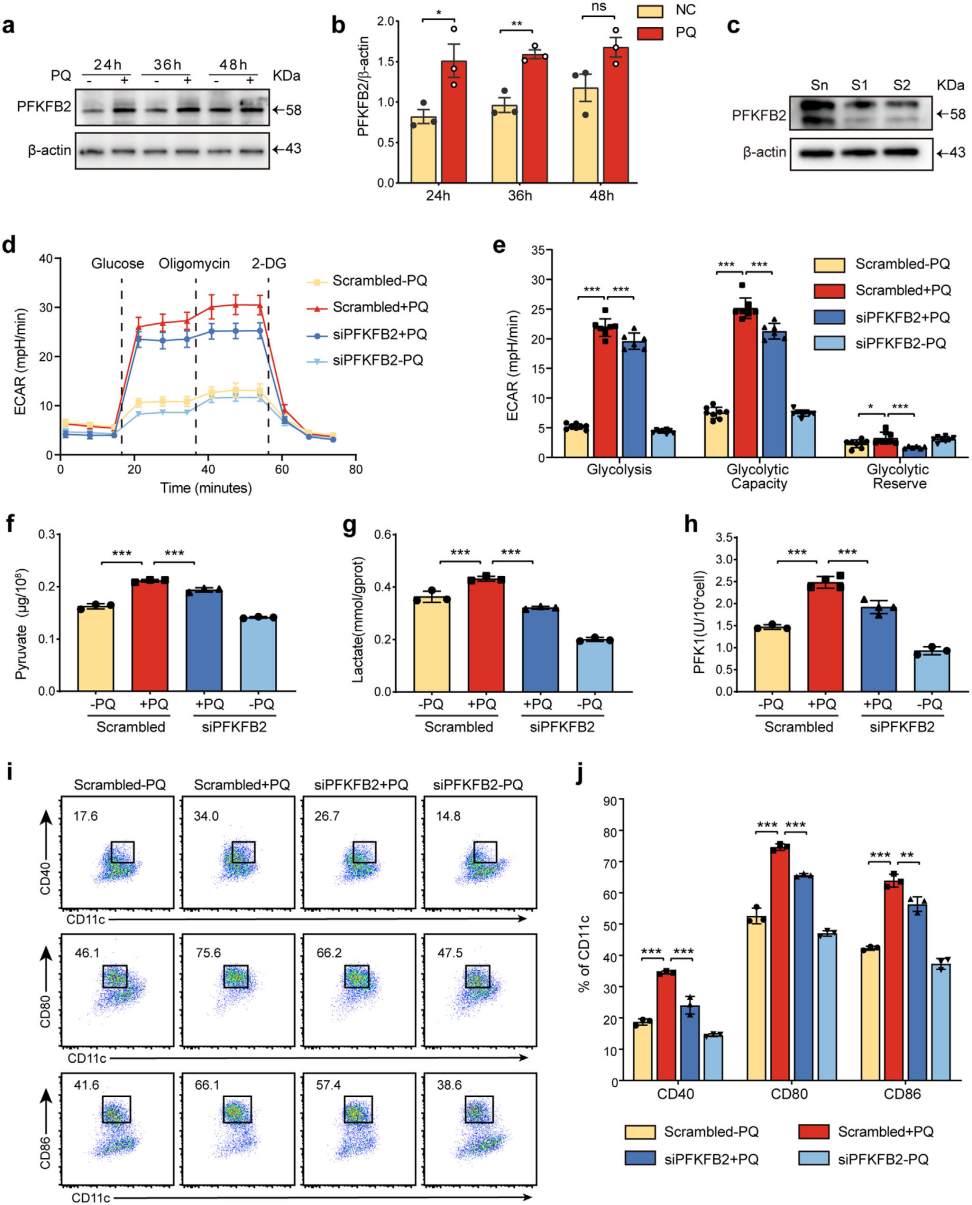
PFKFB2 mediates PQ‐induced glycolysis and BMDC maturation a,b) Western blot analysis of PFKFB2 protein expression levels after treatment with 200 µm PQ for 24, 36, and 48 h (*n* = 3 samples/group). c) Western blot analysis of PFKFB2 expression in DCs after PFKFB2‐siRNA treatment (n = 3 samples/group). d,e) Glycolytic stress test to measure the ECAR of BMDCs (*n* = 6–8 wells/group). f,g) Levels of pyruvate and lactate in BMDCs under different conditions (*n* = 3 wells/group). h) The level of PFK1 enzyme activity (*n* = 3‐4 wells/group). i,j) Flow cytometry detection of BMDC maturation proportion (*n* = 3 wells/group). *^ns^p >* 0.05, ^*^p < 0.05, ^*^ ^*^p < 0.01, ^*^ ^**^p < 0.001. All values are means ± SD, and significance was determined by two‐tailed Student's *t*‐test (b), one‐way ANOVA with Fisher's LSD post hoc analysis (e‐j). Sn: siCon, S1: siPFKFB2‐1, S2: siPFKFB2‐2.

Previous studies have highlighted the important role of glycolysis in DC maturation. Therefore, when further exploring the mechanisms that regulate DC maturation, we paid special attention to the glycolytic pathway. The first rate‐limiting step of glycolysis is catalyzed by PFK1, and the activity of PFK1 is regulated by F2,6BP, which is synthesized and degraded by the PFKFB family of enzymes. We observed that the protein expression level of PFKFB2, a member of this family, was significantly upregulated after PQ treatment (**Figure** **3**a,b). This suggests that PQ may regulate PFK1 activity by up‐regulating PFKFB2 expression, which in turn enhances glycolysis in DCs. To gain a deeper understanding of the role of PFKFB2 in DC glycolysis, we utilized siRNA‐mediated silencing to knock down PFKFB2 in BMDCs (Figure 3c). Real‐time monitoring of the extracellular acidification rate (ECAR) using a glycolytic stress test revealed that the knockdown of PFKFB2 significantly attenuated the glycolytic response, glycolytic capacity, and glycolytic reserve capacity induced by PQ (Figure 3d,e). These findings provide direct evidence that PFKFB2 is essential for the enhanced glycolytic activity observed in PQ‐treated DCs.

Further experiments measured the levels of intracellular pyruvate and lactate, as well as the activity of the key rate‐limiting enzyme PFK1 in glycolysis. We found that the silencing of PFKFB2 reduced the production of glycolytic products and the activity of the rate‐limiting enzyme (Figure 3f–h). These results indicate that PFKFB2 plays an essential role in the glycolytic process induced by PQ. Given the established link between glycolysis and DC maturation, we hypothesized that PFKFB2 may serve as a critical mediator of this process. PFKFB2 knockdown attenuated PQ‐induced BMDC maturation (Figure 3i,j) and shifted cytokine profiles toward an anti‐inflammatory phenotype, as evidenced by decreased TNF‐α, IL‐6, IL‐1β, and increased IL‐10 levels (Figure S3e–h, Supporting Information). In summary, our study reveals the key role of PFKFB2 in promoting DC glycolysis and maturation. PQ enhances DC glycolytic activity by activating PFKFB2, thereby promoting the maturation process. This discovery provides a new perspective for understanding the mechanisms of DC maturation and may offer potential targets for future immune modulation strategies.

### 2.4 PFKFB2 Knockout in DCs Attenuates PQ‐ALI by Suppressing DC Maturation

**Figure 4:**
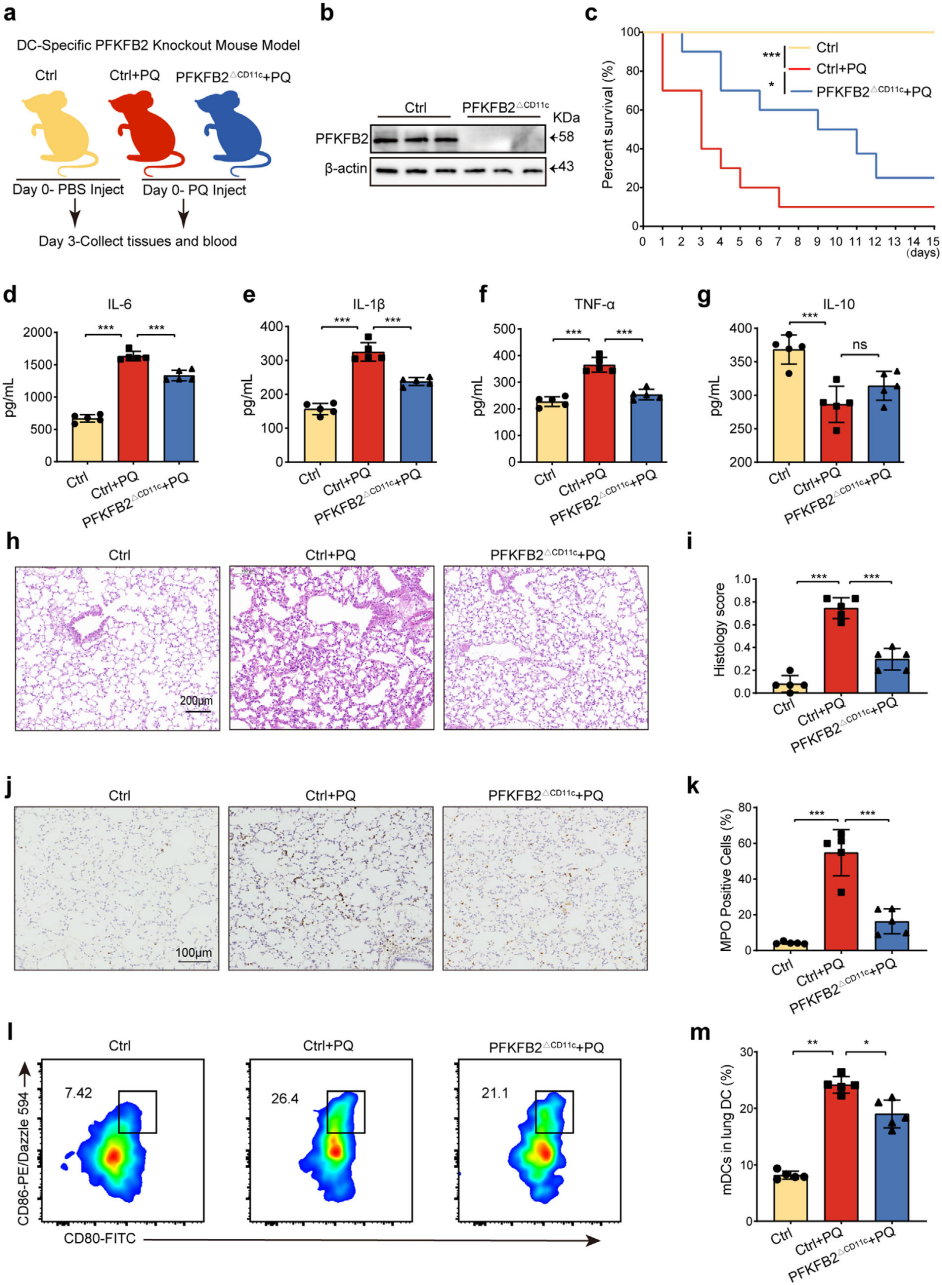
PFKFB2 knockout in DCs mitigates PQ‐ALI by inhibiting DC maturation. a) PFKFB2^fl/fl^ (Ctrl) and PFKFB2^ΔCD11c^ mice received PQ or PBS intraperitoneally. After three days, blood and lung tissues were collected for analysis. b) Western blot detection of PFKFB2 protein levels in BMDCs from WT and PFKFB2^ΔCD11c^ mice (*n* = 3 samples/group). c) The survival curve shows the comparison of survival rates across experimental groups, with PQ administered at a concentration of 40 mg kg^−1^ (*n* = 10 mice/group). d–g) Serum cytokine levels measured by ELISA (*n* = 5 mice/group). h,i) H&E staining and pathological scoring of mouse lung tissue (*n* = 5 mice/group). j,k) Immunohistochemical staining and quantitative results of MPO in lung tissues of mice (*n* = 5 mice/group). l,m) Flow cytometry detection of the maturation proportion of DCs in mouse lungs, with CD80^+^ CD86^+^ cells representing mDCs (*n* = 5 mice/group). *^ns^p >* 0.05, ^*^p < 0.05, ^*^ ^*^p < 0.01, ^*^ ^**^p < 0.001. All values are means ± SD, and significance was determined by Kaplan‐Meier analysis with Log‐rank test (c), or one‐way ANOVA with Fisher's LSD post hoc analysis (d–m).

To clarify the biological role of PFKFB2 in DC maturation and lung injury, we generated a conditional knockout mouse model targeting the PFKFB2 gene specifically in DCs (PFKFB2^ΔCD11c^ mice) and conducted PQ exposure experiments (**Figure** **4**a). Western blot analysis confirmed that PFKFB2 deletion was primarily restricted to DCs by assessing PFKFB2 protein expression in BMDCs and bone marrow‐derived macrophages (BMDMs) from PFKFB2^ΔCD11c^ mice (Figure 4b; Figure S4c,d, Supporting Information). Compared to the PFKFB2^fl/fl^ (Ctrl) group, we observed that the survival rate was significantly reduced by PQ but improved in the PFKFB2^ΔCD11c^ + PQ group (Figure 4c). To evaluate the role of PFKFB2 in DC‐mediated inflammation during PQ‐ALI in vivo, we assessed serum inflammatory cytokines using ELISA. The results showed that, compared to Ctrl + PQ mice, the PFKFB2^ΔCD11c^ + PQ group exhibited significantly lower levels of TNF‐α, IL‐6, and IL‐1β at 72 h, with a slight but non‐significant increase in IL‐10 (Figure 4d–g). Notably, IL‐10 levels became significantly elevated at 96 and 120 h (Figure S5, Supporting Information), indicating that PFKFB2^ΔCD11c^ can effectively suppress PQ‐induced systemic inflammation. H&E staining of lung tissues revealed that the Ctrl group maintained normal lung structure, while the Ctrl + PQ group showed extensive hyaline membrane formation, protein debris filling, diffuse neutrophil infiltration, widespread alveolar hemorrhage, and thickened alveolar walls (Figure 4h,i). In contrast, the PFKFB2^ΔCD11c^ + PQ group exhibited inflammatory cell infiltration and some hyaline membrane formation but no evidence of bleeding or protein debris filling.

To further investigate the inflammatory response, MPO immunohistochemistry was conducted to measure neutrophilic inflammation. MPO activity was significantly elevated in the lung tissues of Ctrl + PQ mice compared to Ctrl mice, whereas MPO activity was markedly reduced in PFKFB2^ΔCD11c^ + PQ mice (Figure 4j,k), suggesting that PFKFB2^ΔCD11c^ mitigates PQ‐induced lung damage and inflammation. Additionally, we observed that PQ treatment increased the proportion of mature pulmonary DCs in mice, but PFKFB2‐specific knockout led to a significant reduction in DC maturation in the PFKFB2^ΔCD11c^ + PQ group (Figure 4l,m). BMDCs from Ctrl and PFKFB2^ΔCD11c^ mice were extracted for in vitro validation (Figure S2a, Supporting Information). After PQ treatment, the maturation proportion of PFKFB2^ΔCD11c^ BMDCs was significantly lower than that of the Ctrl group (Figure S2b,c, Supporting Information). In summary, PQ may promote DC maturation through PFKFB2, thereby exacerbating lung injury.

### 2.5 PQ Promotes DC Glycolysis and Maturation by Regulating PFKFB2 via HIF‐1α

**Figure 5:**
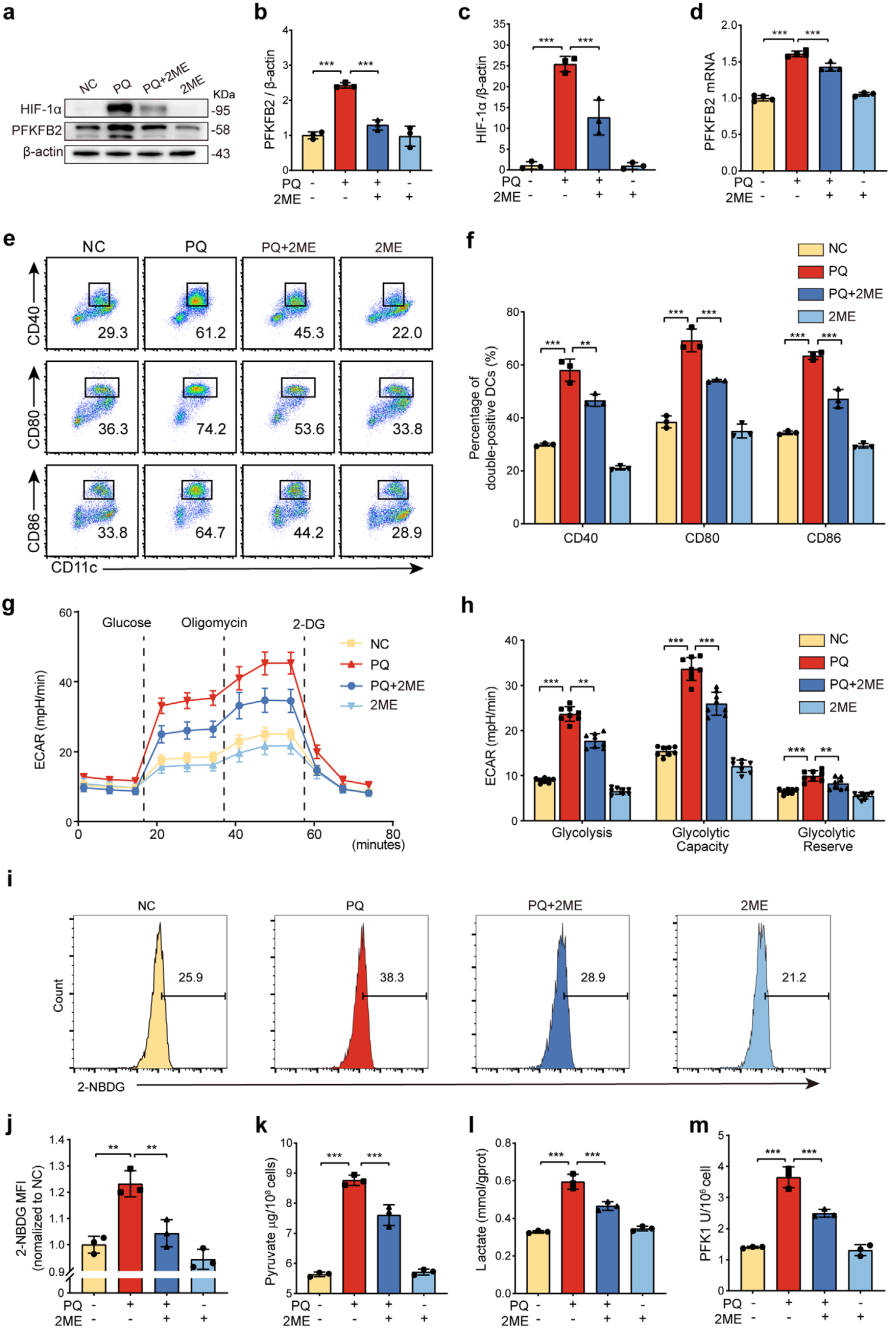
HIF‐1α mediates PQ‐induced upregulation of PFKFB2, promoting DC glycolysis and maturation. a‐c) Western blot analysis of PFKFB2 and HIF‐1α protein levels in BMDCs treated with 200 µm PQ and 2 µm 2ME for 24 h (*n* = 3 samples/group). d) qRT‐PCR detection of HIF‐1α mRNA levels in BMDCs (n = 3‐4 wells/group). e,f) Flow cytometry detection of BMDC maturation proportion (*n* = 3 wells/group). g,h) Glycolytic stress test showing extracellular acidification rate (ECAR) in BMDCs (*n* = 8 wells/group). i,j) Flow cytometry analysis of glucose uptake using the fluorescent glucose analog 2‐NBDG (*n* = 3 wells/group). k,l) Levels of pyruvate and lactate in BMDCs under different conditions (*n* = 3 wells/group). m) The level of PFK1 enzyme activity (*n* = 3 wells/group). ^*^ ^*^p < 0.01, ^*^ ^**^p < 0.001. All values are means ± SD, and significance was determined by one‐way ANOVA with Fisher's LSD post hoc analysis.

To investigate whether PQ regulates PFKFB2 expression via HIF‐1α to influence DC glycolysis and maturation, BMDCs were treated with the HIF‐1α inhibitor 2‐methoxyestradiol (2ME). A cytotoxicity assay identified 2 µm as the optimal concentration for 24 h treatment (Figure S6a, Supporting Information). PQ treatment significantly increased both protein and mRNA levels of HIF‐1α, while 2ME treatment markedly suppressed PFKFB2 expression (**Figure** **5**a–d; Figure S6b,c, Supporting Information). These results indicate that PQ‐induced upregulation of PFKFB2 is mediated, at least in part, by HIF‐1α.

Further analysis using flow cytometry demonstrated that 2ME markedly inhibited PQ‐induced BMDC maturation and pro‐inflammatory cytokine production, while elevating IL‐10 levels (Figure 5e,f; Figure S3i–l, Supporting Information). This finding underscores the role of HIF‐1α in promoting DC maturation under PQ stimulation. To explore the metabolic basis of this process, glycolytic parameters were assessed. PQ treatment enhanced glycolysis in BMDCs, as indicated by increased ECAR, glucose uptake, glycolytic products (pyruvate and lactate), and the activity of the rate‐limiting glycolytic enzyme PFK1. However, these glycolytic parameters were significantly reduced following 2ME treatment (Figure 5g–m). Collectively, these results demonstrate that PQ enhances DC glycolysis and maturation by upregulating PFKFB2 expression through HIF‐1α.

### 2.6 HIF‐1α Directly Binds to the PFKFB2 Promoter to Regulate Its Transcription in DCs

**Figure 6:**
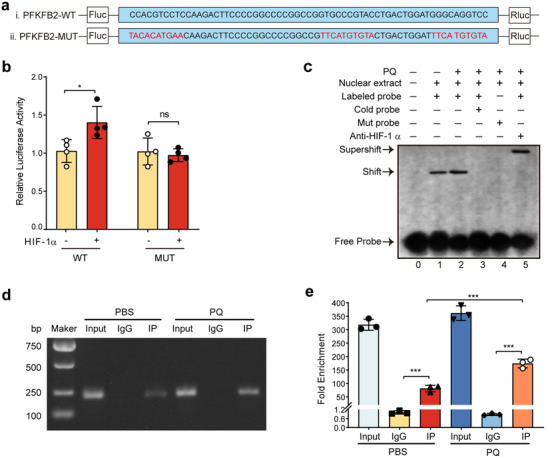
Molecular mechanism of HIF‐1α binding to the PFKFB2 promoter. a) Schematic representation of the WT and MUT PFKFB2 promoter constructs used for the dual‐luciferase reporter assay, highlighting the predicted HIF‐1α binding site. b) These fragments were inserted into the pGL3‐Basic vector and transfected into 293T cells with lipofectamine 3000, along with the expression vector containing the HIF‐1α transcription factor. After 24 h of transfection, the reporter gene assay was conducted (*n* = 4 wells/group). c) EMSA results: Lane 1: No competition probe; Lane 2: PQ intervention, no competition probe; Lane 3: Wild‐type cold probe competition; Lane 4: Mutated probe competition; Lane 5: HIF‐1α antibody. d,e) ChIP results and enrichment analysis (*n* = 3 samples/group). *^ns^p >* 0.05, ^*^p < 0.05, ^*^ ^**^p < 0.001. All values are means ±  SD, and significance was determined by two‐tailed Student's *t*‐test (b), one‐way ANOVA with Fisher's LSD post hoc analysis (e). WT: wild type, MUT: mutant type.

Under hypoxic conditions, HIF‐1α has been reported to upregulate PFKFB2 expression through direct transcriptional regulation, though this mechanism may vary depending on cell type.^[^ ^19^ ^]^ To investigate the molecular mechanism by which HIF‐1α regulates PFKFB2 transcription in DCs, dual‐luciferase reporter assays were employed to identify the active binding regions of HIF‐1α on the PFKFB2 promoter. Using the JASPAR database, potential HIF‐1α binding sites on the PFKFB2 promoter were predicted. Subsequently, wild‐type (WT) and mutant‐type (MUT) dual‐luciferase reporter constructs were generated for further analysis (**Figure** **6**a). The dual‐luciferase assay revealed that HIF‐1α overexpression significantly increased luciferase activity in cells transfected with the WT PFKFB2 promoter construct, whereas no significant change was observed in cells transfected with the MUT construct (Figure 6b). These results suggest that HIF‐1α directly regulates PFKFB2 transcription by binding to specific promoter regions.

To validate this interaction, electrophoretic mobility shift assays (EMSA) were performed. Nuclear extracts from BMDCs exposed to either PBS or PQ were incubated with a probe containing the WT sequence of the PFKFB2 promoter. The results demonstrated that the nuclear extracts bound specifically to the WT probe, while no binding was observed with the MUT probe (Figure 6c). The specificity of this interaction was further confirmed by competition assays using an unlabeled cold probe, which abolished binding, and by the addition of an anti‐HIF‐1α antibody, which caused a supershift. Chromatin immunoprecipitation (ChIP) assays provided additional evidence supporting the direct interaction between HIF‐1α and the PFKFB2 promoter. The results showed that HIF‐1α binding to the PFKFB2 promoter was significantly enriched in PQ‐treated BMDCs compared to controls (Figure 6d,e). These findings align with the results from the dual‐luciferase and EMSA assays, demonstrating that HIF‐1α directly regulates PFKFB2 transcription by binding to its promoter region and that PQ treatment enhances this interaction.

### 2.7 Inhibition of the HIF‐1α–PFKFB2 Signaling Pathway in Pulmonary DCs Attenuates PQ‐ALI

**Figure 7:**
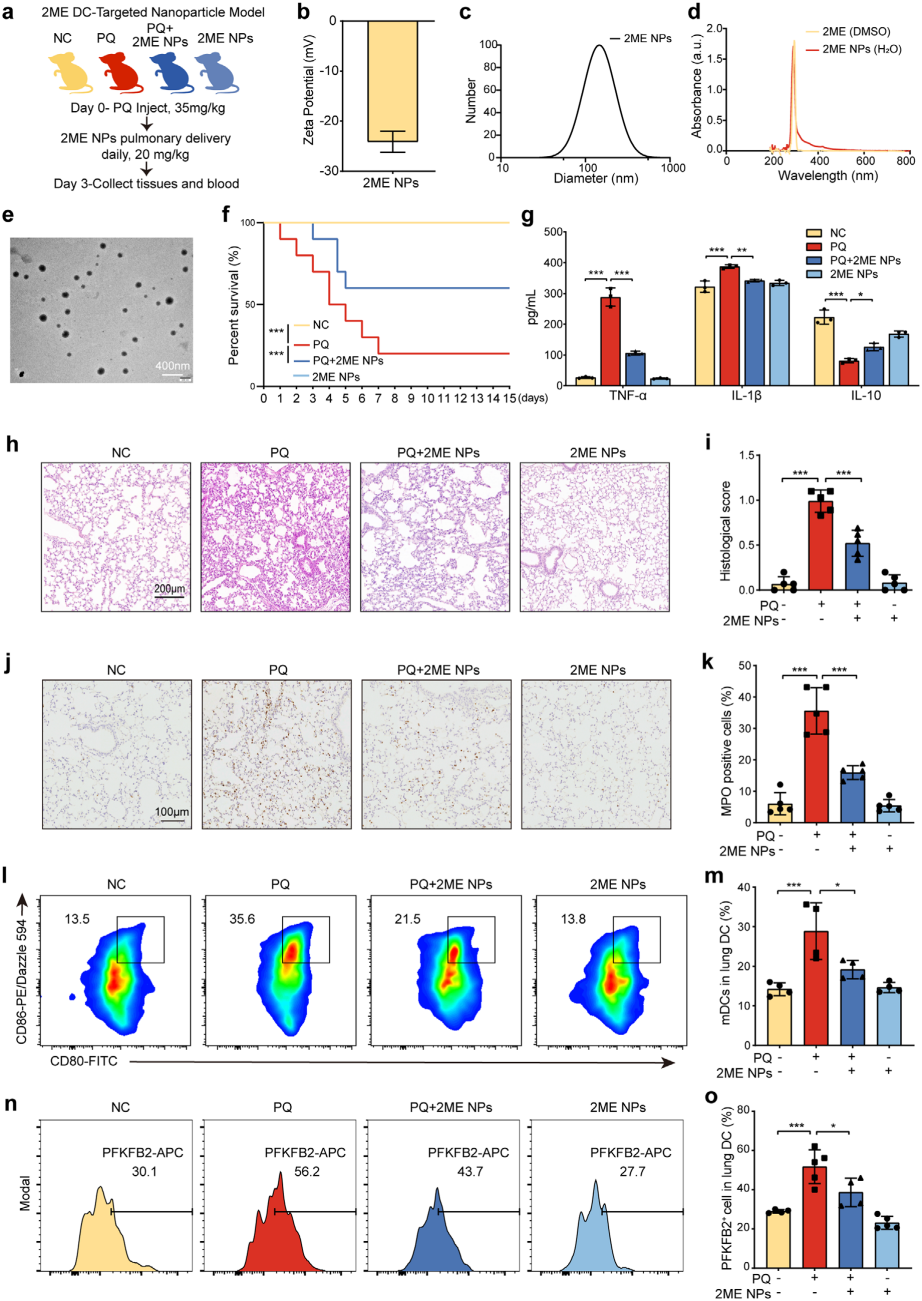
Nanoparticle‐mediated targeting of HIF‐1α in DCs mitigates PQ‐ALI by modulating the HIF‐1α‐PFKFB2 signaling pathway. a) Experimental design created with figdraw.com. Mice received PQ or PBS intraperitoneally, along with 2ME NPs via intratracheal delivery daily. After three days, blood and lung tissues were collected for analysis. b–e) Analysis of Zeta potential, particle size, UV–vis absorption spectrum, and transmission electron microscopy of 2ME NPs (*n* = 3 samples/group). f) The survival curve shows the comparison of survival rates across experimental groups, with PQ administered at a concentration of 40 mg kg^−1^ (*n* = 10 mice/group). g) The levels of TNF‐α, IL‐1β, and IL‐10 in mouse serum were detected using ELISA (*n* = 3 mice/group). h,i) Lung H&E staining and pathological scoring (*n* = 5 mice/group). j,k) Immunohistochemical staining and quantitative results of MPO in lung tissues of mice (*n* = 5 mice/group). l,m) Flow cytometry detection of the maturation proportion of DCs in mouse lungs, with CD80^+^ CD86^+^ cells representing mDCs (*n* = 4 mice/group). n,o) Flow cytometry detection of PFKFB2 expression in mouse pulmonary DCs (*n* = 5 mice/group). ^*^p < 0.05, ^*^ ^*^p < 0.01, ^*^ ^**^p < 0.001. All values are means ±  SD, and significance was determined by one‐way ANOVA with Fisher's LSD post hoc analysis (g‐o) or Kaplan–Meier analysis with Log‐rank test (f). 2ME NPs: 2‐methoxyestradiol@DSPE‐PEG‐MAN nanoparticles.

To investigate whether HIF‐1α contributes to lung injury through DCs in mice, a DC‐targeted HIF‐1α inhibitor was developed and evaluated in vivo (**Figure** **7**a). DC targeting, an approach in vaccine delivery, enhances drug concentration at the target site, thereby improving therapeutic efficacy while minimizing systemic side effects. In this study, mannose was employed as a modifying molecule to achieve DC targeting, and 2‐methoxyestradiol@DSPE‐PEG‐MAN nanoparticles (2ME NPs) were synthesized using the film hydration method with mannose‐modified PEG‐DSPE and 2ME. Comprehensive characterization of the nanoparticles demonstrated a Zeta potential of ‐24.1 ± 0.997 mV (Figure 7b) and a particle size of 130.44 ± 3.50 nm (Figure 7c), indicating stability and uniformity in solution. UV–vis absorption spectroscopy confirmed the preservation of the structural integrity of 2ME during encapsulation (Figure 7d), while transmission electron microscopy further validated the uniformity of the nanoparticles (Figure 7e). The in vitro drug release profile of 2ME from DSPE‐PEG‐MAN nanoparticles was evaluated using the dialysis bag method. As shown in the release curve, in the absence of phospholipase, the nanoparticles exhibited good stability, with only ≈30% cumulative drug release over 24 h. In contrast, in the presence of phospholipase, over 90% of 2ME was released within the same period, with a rapid initial burst release observed during the first 6 h. These results indicate that the nanoparticles are highly responsive to phospholipase, showing minimal drug release under normal physiological conditions but undergoing efficient drug release when exposed to phospholipase‐rich environments such as lysosomes (Figure S7a, Supporting Information).

The colloidal stability of the nanoparticles was evaluated by monitoring their hydrodynamic diameter and zeta potential over a 7‐day period at 4  °C. The particle size remained relatively constant throughout the storage period, with less than 5% variation, indicating no significant aggregation or degradation. Similarly, the zeta potential values fluctuated within a narrow range (± 2 mV), further confirming the stability of the nanoparticle surface charge. These findings demonstrate that the nanoparticles possess excellent colloidal stability under the tested conditions (Figure S7b,c, Supporting Information). In addition, we conducted biosafety evaluations in both mouse and cell models. H&E staining, pathological scoring, hematological parameters, and hepatic and renal function tests revealed no significant differences among the NC, 2ME NPs, and nanoparticles without 2ME loading (Ctrl NPs) groups, indicating no detectable toxicity to the lungs, liver, kidneys, heart, and spleen (Figure S8a–j and Table S1, Supporting Information). In vitro CCK‐8 assays further demonstrated that DSPE‐PEG‐MAN nanoparticles, with or without 2ME, had no significant impact on the viability of MLE‐12, BEAS‐2B, or BMDCs (Figure S8k–m, Supporting Information). Collectively, these findings confirm the favorable biocompatibility of the nanoparticle formulation under both in vivo and in vitro conditions.

Nanomedicines are frequently employed in the treatment of ALI via pulmonary or intravenous administration.^[^ ^26^ ^]^ In ALI, elevated lung permeability facilitates passive targeting of nanomedicines through pulmonary delivery.^[^ ^27^ ^]^ In this study, 2ME NPs were administered intratracheally to deliver the HIF‐1α inhibitor directly to lung DC. To confirm effective delivery, DIR‐labeled nanoparticles were administered intratracheally in mice and tracked using IVIS at 0, 1, 24, 48, and 72 h. Lung fluorescence intensity increased initially and then declined over time (Figure S9a, Supporting Information). Quantitative analysis confirmed predominant lung accumulation of 2ME NPs‐DIR, with no significant differences between PQ‐treated and control mice (Figure S9b, Supporting Information). Ex vivo imaging further verified lung‐specific distribution without notable accumulation in other major organs (Figure S9c,d, Supporting Information). Additionally, to assess DC‐targeting efficiency, Cy5.5‐labeled nanoparticles were incubated with BMDCs and MLE‐12 cells. Flow cytometry showed markedly higher uptake in BMDCs, confirming selective targeting (Figure S10a,b, Supporting Information). Immunofluorescence of lung sections further demonstrated strong colocalization of CD11c with DIR‐labeled NPs, validating in vivo DC targeting (Figure S10c, Supporting Information). These findings demonstrate that the nanoparticles were successfully delivered to the lungs via intratracheal administration and effectively targeted lung DC, thereby establishing a foundation for subsequent pharmacodynamic studies and potential clinical applications.

In subsequent in vivo experiments, mice were divided into four groups: NC group (PBS + Ctrl NPs), PQ group (PQ + Ctrl NPs), PQ + 2ME NPs group, and 2ME NPs group (PBS + 2ME NPs). The survival rate of the PQ + 2ME NPs group was significantly higher than that of the PQ group (Figure 7f). ELISA analysis revealed that inflammatory cytokine levels in the serum, including TNF‐α and IL‐1β, were significantly reduced in the PQ + 2ME NPs group, while the anti‐inflammatory cytokine IL‐10 was markedly increased (Figure 7g). Histopathological analysis of lung tissues corroborated these findings. The NC group exhibited normal lung architecture, whereas the PQ group displayed severe pathological changes, including diffuse neutrophil infiltration, widespread alveolar hemorrhage, extensive hyaline membrane formation, proteinaceous debris accumulation, and thickened alveolar walls. In contrast, the PQ + 2ME NPs group showed reduced inflammatory cell infiltration, partial hyaline membrane formation, and moderate alveolar wall thickening, with no evidence of hemorrhage or proteinaceous debris accumulation (Figure 7h,i). Furthermore, MPO immunohistochemistry revealed that MPO activity, which was significantly elevated in the PQ group, was markedly reduced in the PQ + 2ME NPs group (Figure 7j,k). Flow cytometry analysis provided additional insights into the mechanism of action. PQ‐induced DC maturation, characterized by increased CD80^+^CD86^+^ cells, was significantly inhibited by 2ME NPs (Figure 7l,m). Additionally, flow cytometry analysis of PFKFB2 expression in lung DC demonstrated that PQ treatment upregulated PFKFB2 expression, whereas 2ME NPs effectively downregulated PFKFB2 levels (Figure 7n,o). These results suggest that PQ promotes lung DC glycolysis and maturation via the HIF‐1α‐PFKFB2 signaling pathway, ultimately leading to lung injury.

To further explore the impact of PQ on the immune microenvironment, we analyzed the proportions of regulatory T cells (Tregs) and T helper 1 (Th1) cells in the lungs of PQ‐ALI mouse models. PQ treatment led to a significant reduction in the proportion of Tregs, while the proportion of Th1 cells remained unchanged, resulting in a decreased Treg/Th1 ratio. Notably, treatment with the DC‐targeted 2ME nanoparticles restored Treg proportions and rebalanced the Treg/Th1 ratio (Figure S11a–e, Supporting Information). Furthermore, in the in vitro mixed lymphocyte reaction, we observed that after co‐culturing PQ‐pretreated BMDCs with T cells for 24 h, the proportion of CD4⁺CD25⁺ cells in T cells was significantly increased, while 2ME intervention significantly reversed this change (Figure S11f,g, Supporting Information). These results demonstrate that PQ promotes DC maturation, leading to Treg/Th1 imbalance and local immune disturbances, potentially contributing to the progression of lung injury. Furthermore, these findings highlight the critical role of DCs as key regulators in developing therapeutic strategies for lung injury treatment.

### 2.8 PFKFB2‐Mediated Maturation of Pulmonary DCs Promotes LPS‐ALI

**Figure 8:**
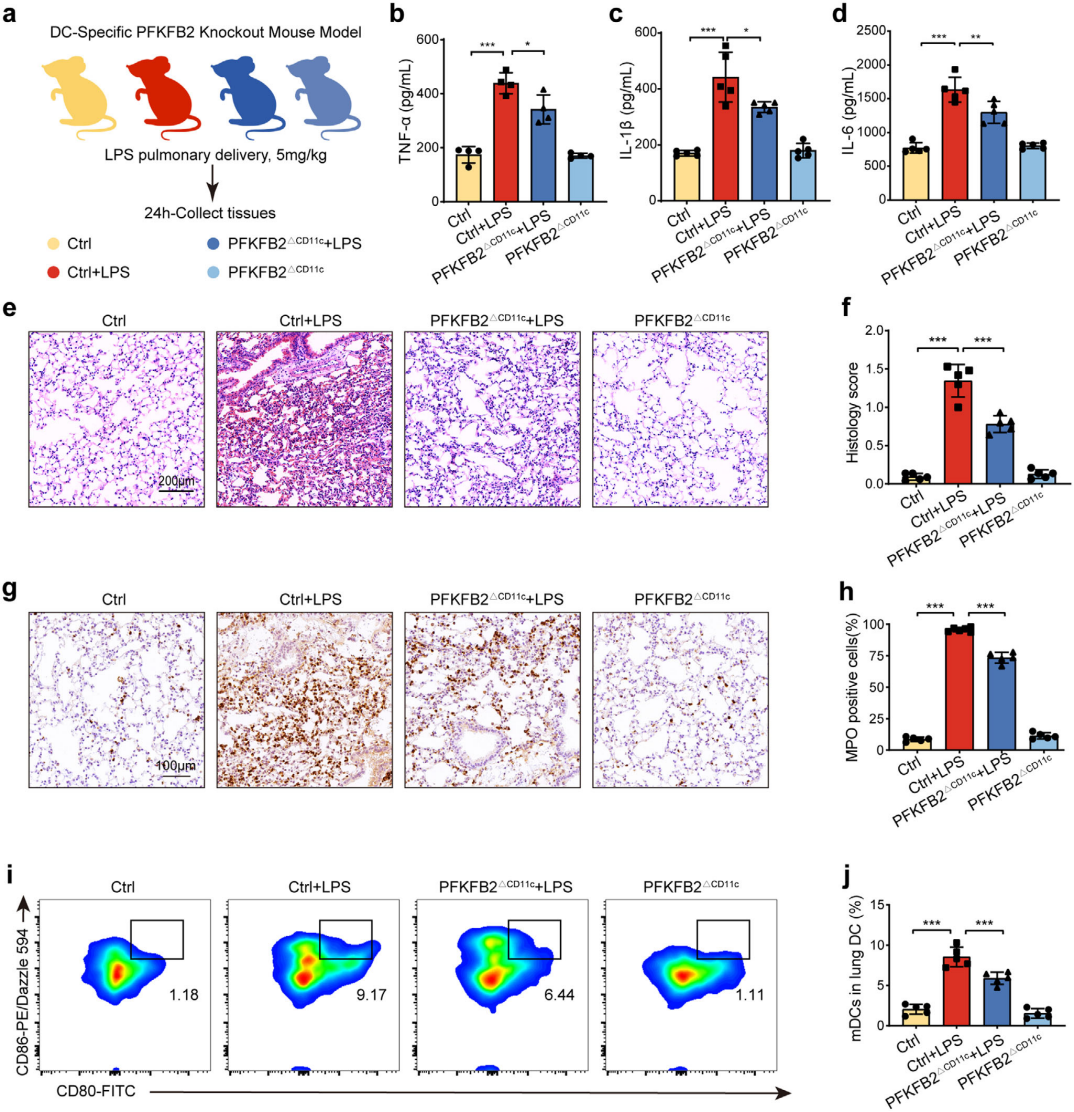
PFKFB2 knockout in DCs mitigates LPS‐ALI by inhibiting DC maturation. a) Experimental design, created with figdraw.com. PFKFB2^fl/fl^ and PFKFB2^ΔCD11c^ mice received LPS or PBS intraperitoneally. After 24 h, blood and lung tissues were collected for analysis. b–d) ELISA detection of TNF‐α, IL‐1β, and IL‐6 levels in the serum of mice from each group (*n* = 4‐5 mice/group). e,f) H&E staining and pathological scoring of mouse lung tissue (*n* = 5 mice/group). g,h) Immunohistochemical staining and quantitative results of MPO in lung tissues of mice (*n* = 5 mice/group). i,j) Flow cytometry detection of the maturation proportion of DCs in mouse lungs, with CD80^+^ CD86^+^ cells representing mDCs (*n* = 5 mice/group). ^*^p < 0.05, ^*^ ^*^p < 0.01, ^*^ ^**^p < 0.001. All values are means ± SD, and significance was determined by one‐way ANOVA with Fisher's LSD post hoc analysis.

To investigate whether PFKFB2 contributes to lung injury induced by factors beyond PQ, LPS, a well‐established inducer of ALI, was utilized to create a bilateral pulmonary inflammation model in mice. This study employed PFKFB2 conditional knockout mice (PFKFB2^ΔCD11c^) to evaluate the role of PFKFB2 in LPS‐ALI (**Figure** **8**a). Mice were divided into four experimental groups: the control group (PFKFB2^fl/fl^ + PBS), the LPS group (PFKFB2^fl/fl^ + LPS), the PFKFB2^ΔCD11c^ + LPS group, and the PFKFB2^ΔCD11c^ group (PFKFB2^ΔCD11c^ + PBS). Compared to the LPS group, PFKFB2^ΔCD11c^ + LPS mice exhibited significantly reduced serum levels of pro‐inflammatory cytokines, including TNF‐α, IL‐6, and IL‐1β (Figure 8b–d), suggesting that PFKFB2 knockout alleviated LPS‐induced inflammation. Histopathology showed decreased neutrophil infiltration, reduced hyaline membrane formation, and less pronounced alveolar wall thickening in PFKFB2^ΔCD11c^ + LPS mice compared to their LPS‐treated counterparts (Figure 8e,f). Immunohistochemical staining revealed that elevated MPO activity in LPS mice was attenuated in PFKFB2^ΔCD11c^ + LPS mice (Figure 8g,h). Flow cytometry analysis provided additional insights into the role of PFKFB2 in lung DC maturation. LPS exposure significantly increased the proportion of mDCs. However, this effect was markedly inhibited in PFKFB2^ΔCD11c^ + LPS mice (Figure 8i,j). These findings demonstrate that LPS promotes DC maturation through PFKFB2, contributing to the pathogenesis of ALI.

### 2.9 Pulmonary DC‐Specific Inhibition of HIF‐1α‐PFKFB2 Signaling Mitigates LPS‐ALI

**Figure 9:**
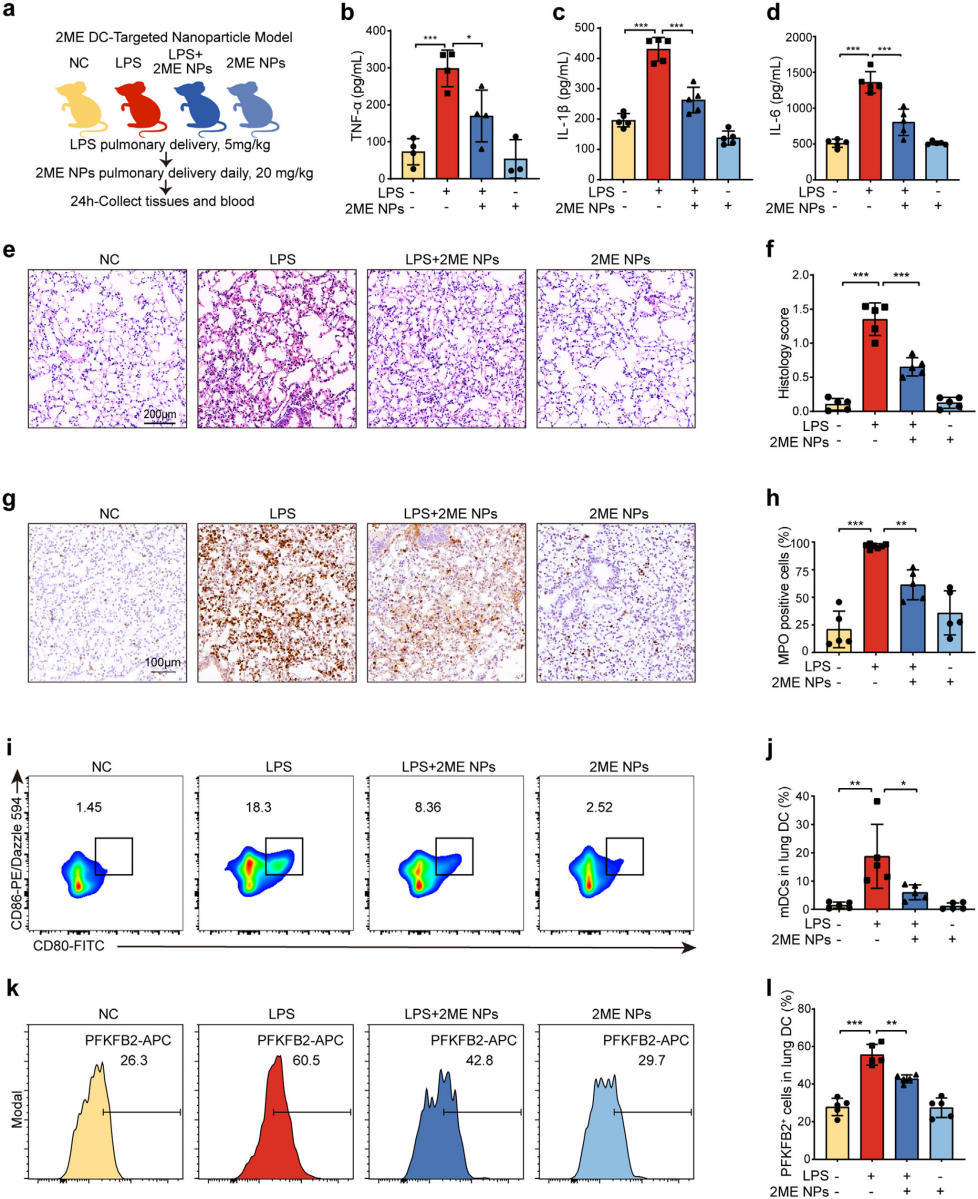
Nanoparticle‐mediated targeting of HIF‐1α in DCs mitigates LPS‐ALI by modulating the HIF‐1α‐PFKFB2 signaling pathway. a) Experimental design created with figdraw.com. Mice received LPS or PBS intraperitoneally, along with 2ME NPs via intratracheal delivery daily, and the mice's blood and lung tissues were collected for analysis after 24 h. b–d) ELISA detection of TNF‐α, IL‐1β, and IL‐6 levels in the serum of mice (*n* = 3–5 mice/group). e,f) H&E staining and pathological scoring of mouse lung tissue (*n* = 5 mice/group). g,h) Immunohistochemical staining and quantitative results of MPO in lung tissues of mice (*n* = 5 mice/group). i,j) Flow cytometry detection of the maturation proportion of DCs in mouse lungs, with CD80^+^CD86^+^ cells representing mDCs (*n* = 5 mice/group). k,l) Flow cytometric measurement of PFKFB2 expression in mouse lung DCs (*n* = 4 mice/group). ^*^p < 0.05, ^*^ ^*^p < 0.01, ^*^ ^**^p < 0.001. All values are means ±  SD, and significance was determined by one‐way ANOVA with Fisher's LSD post hoc analysis. 2ME NPs: 2‐methoxyestradiol@DSPE‐PEG‐MAN nanoparticles.

To evaluate the therapeutic potential of targeting the HIF‐1α‐PFKFB2 signaling pathway in DCs, this study investigated the efficacy of a DC‐targeted HIF‐1α inhibitor, 2ME NPs, in mitigating LPS‐induced ALI in a mouse model (**Figure** **9**a). Mice treated with both LPS and 2ME NPs exhibited significantly reduced serum levels of pro‐inflammatory cytokines, including TNF‐α (Figure 9b), IL‐1β (Figure 9c), and IL‐6 (Figure 9d). Histopathological analysis showed clear differences between groups. NC mice had intact lung architecture with no inflammation, while LPS‐treated mice displayed severe lung injury, including neutrophil infiltration, hyaline membranes, alveolar hemorrhage, and thickened walls. In contrast, LPS + 2ME NPs‐treated mice showed improved pathology, with reduced cell infiltration, partial hyaline membrane resolution, and moderate wall thickening, but no alveolar hemorrhage or proteinaceous debris (Figure 9e,f). Furthermore, MPO immunohistochemistry revealed that MPO activity, which was significantly elevated in the LPS group, was markedly reduced in the LPS + 2ME NPs group (Figure 9g,h), further confirming the anti‐inflammatory effects of 2ME NPs.

To assess the impact of the treatment on DC maturation, flow cytometry analysis was performed. LPS exposure significantly increased the maturation of lung DC. However, treatment with 2ME NPs significantly inhibited DC maturation, demonstrating the capacity of these nanoparticles to suppress LPS‐induced DC activation (Figure 9i,j). Additionally, flow cytometry analysis of PFKFB2 expression in lung DC demonstrated that LPS treatment upregulated PFKFB2 expression, whereas 2ME NPs effectively downregulated PFKFB2 levels (Figure 9k,i). In conclusion, this study provides compelling evidence for the therapeutic potential of DC‐targeted HIF‐1α inhibitors, such as 2ME NPs, in treating ALI. By modulating the HIF‐1α‐PFKFB2 signaling pathway, these nanoparticles effectively suppressed inflammation and DC maturation, thereby alleviating lung injury. This approach offers a promising avenue for the development of targeted therapies for ALI and other inflammatory lung diseases.

### 2.10 Enhanced Pulmonary DC Maturation and PFKFB2 Expression in Patients with PQ Poisoning, 2°BP and COVID‐19

**Figure 10:**
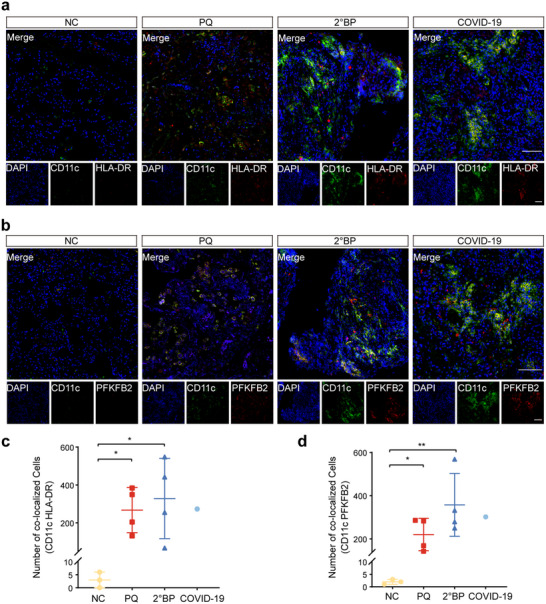
Enhanced maturation of pulmonary dendritic cells and upregulated PFKFB2 expression in lung tissues from patients with PQ poisoning, 2°BP, and COVID‐19. a,c) Double immunofluorescence labeling with CD11c and HLA‐DR shows enhanced maturation of pulmonary DCs in lung tissues from patients with PQ poisoning, 2°BP, and COVID‐19. (NC: *n* = 3; PQ: *n* = 4; 2°BP: *n* = 4; COVID‐19: *n* = 1). b,d) Co‐immunofluorescence staining with CD11c and PFKFB2 demonstrates elevated PFKFB2 expression in pulmonary DCs within lung tissues of patients with PQ poisoning, 2°BP, and COVID‐19. (NC: *n* = 3; PQ: *n* = 4; 2°BP: *n* = 4; COVID‐19: *n* = 1). ^*^p < 0.05, ^*^ ^*^p < 0.01. All values are means ±  SD, and significance was determined by one‐way ANOVA with Fisher's LSD post hoc analysis. 2°BP: secondary bacterial pneumonia. COVID‐19: Coronavirus Disease 2019.

Our previous studies using BMDCs cell models, as well as PQ‐ALI and LPS‐ALI animal models, revealed that PQ and LPS aggravate ALI by enhancing lung DC maturation through the upregulation of PFKFB2 expression. To validate these findings in a clinical context, we collected lung tissue samples from patients with ALI caused by different etiologies, including 4 cases of PQ poisoning patients (toxic origin), 4 cases of 2°BP (bacterial origin), and 1 case of COVID‐19 patient (viral origin), along with 3 normal controls (NC). Double immunofluorescence labeling with CD11c and HLA‐DR revealed an increase in the number of cells co‐expressing CD11c and HLA‐DR in lung tissues from the three patient groups, suggesting elevated levels of DC maturation (**Figure** **10**a,c). Furthermore, double labeling with CD11c and PFKFB2 showed higher PFKFB2 expression in DCs from the lung tissues of these three patient groups compared to NCs (Figure 10b,d). Although the COVID‐19 group includes only one sample, the observed trend is consistent with those of the PQ and 2°BP group, supporting the relevance of these findings. Peripheral blood transcriptomic analysis further validated these findings. The RNA sequencing results showed that PFKFB2 expression was significantly upregulated in the peripheral blood of PQ‐exposed patients compared to NCs (Figure S12 and Table S2, Supporting Information). The volcano plot highlights PFKFB2 as one of the most upregulated genes, underscoring its potential role in systemic immune responses associated with PQ exposure. These findings align with results from animal experiments. Constrained by the limited sample size and imbalanced distribution (particularly among COVID‐19 cases), our data preliminarily suggest that the PFKFB2‐mediated DC maturation mechanism may be conserved across diverse etiologies of ALI. Future studies with expanded cohorts are warranted to validate its clinical translational potential.

## 3 Discussion

Over the past few decades, mounting evidence has established a strong link between the intracellular metabolism of immune cells and the regulation of inflammatory responses. For instance, studies have demonstrated that macrophage‐targeted nanoplatforms, combined with inhalation delivery systems, effectively downregulate HK2, a critical metabolic and inflammatory regulatory factor, in lung macrophages, alleviating LPS‐ALI in mice. These findings underscore the pivotal role of immune cell metabolism in modulating inflammation.^[^ ^27^ ^]^ Building upon this foundation, our study provides novel insights into the metabolic mechanisms underlying inflammatory diseases. Specifically, our results demonstrated that HIF‐1α directly binds to the promoter region of PFKFB2, thereby transcriptionally activating its expression and enhancing glycolytic activity. This metabolic reprogramming promotes DC maturation, ultimately aggravating inflammation and lung injury. Notably, specific knockout of PFKFB2 in DCs or targeted inhibition of HIF‐1α in DCs significantly mitigated ALI. Furthermore, the conservation of this metabolic regulatory axis in human lung diseases highlights its clinical relevance and translational potential. Collectively, these findings illuminate the intricate relationship between immune metabolism and inflammatory imbalance, offering promising therapeutic targets for ALI.

A hallmark feature of ALI is the infiltration and migration of inflammatory cells into the damaged pulmonary interstitium. DCs, as key antigen‐presenting cells, play a central role in orchestrating the inflammatory response. During lung injury, DCs express co‐stimulatory molecules such as CD86 and CD80, rapidly accumulate in the pulmonary interstitium, and release a cascade of inflammatory mediators, thereby amplifying lung injury.^[^ ^8^, ^9^, ^28^ ^]^ Our study corroborated these observations by demonstrating that lung DC maturation significantly increased in a PQ‐ALI mouse model and remained elevated even five days post‐ALI induction. In vitro experiments with BMDCs further validated this maturation phenotype. These findings suggest that modulating DC maturation represents a critical therapeutic approach for ALI. However, the specific mechanisms and regulatory pathways governing DCs behavior in the ALI microenvironment remain incompletely understood, warranting further investigation.

Glycolysis plays an indispensable role in DC maturation by providing the energy required to sustain their activation and function. While oxidative phosphorylation is the primary and most efficient ATP production pathway, glycolysis becomes the preferred metabolic route for cells with high energy demands, such as maturing DCs. Consistent with prior studies ^[^ ^29^, ^30^ ^]^ our findings revealed a significant increase in glucose uptake and glycolytic activity in BMDCs during PQ‐induced maturation. Glycolytic inhibitors effectively suppressed DC maturation, further underscoring the necessity of glycolysis in this process.^[^ ^31^ ^]^ Previous studies have indicated that glycolytic metabolites such as lactate can suppress mtROS‐ and XBP1‐driven pro‐inflammatory responses in DCs during autoimmunity ^[^ ^32^ ^]^ while pyruvate is essential for type I interferon responses in plasmacytoid DCs.^[^ ^33^ ^]^ In our ALI model, both PFKFB2 knockdown and HIF‐1α inhibitor intervention reduced glycolytic metabolites and correspondingly attenuated DC maturation, suggesting potential mediation by these metabolites. However, this study has not established direct causal links between specific metabolites and DC maturation, such as through rescue of maturation defects via exogenous metabolite supplementation. Future investigations should further examine whether glycolytic metabolites directly drive DC maturation in ALI contexts.

The glycolytic regulator PFKFB2 promotes neutrophil inflammation during sepsis.^[^ ^16^ ^]^ and sustains lactate‐driven macrophage phagocytosis.^[^ ^18^ ^]^ Our findings demonstrate that PFKFB2‐mediated glycolysis drives BMDC maturation, enhancing secretion of pro‐inflammatory cytokines (TNF‐α, IL‐1β, IL‐6) while suppressing anti‐inflammatory IL‐10 production. In both PQ and LPS‐induced ALI models, we observed significantly increased pulmonary DC maturation and elevated pro‐inflammatory cytokines. DC‐specific conditional knockout of PFKFB2 reduced pulmonary DC maturation proportions, decreased pro‐inflammatory cytokine levels, and attenuated lung injury, indicating that toxin/pathogen‐induced PFKFB2 upregulation promotes pulmonary DC maturation, thereby exacerbating ALI progression. Notably, PFKFB2 knockout in DCs significantly reduced serum pro‐inflammatory cytokines in ALI mice at 72 h post‐induction, while IL‐10 showed a non‐significant increasing trend. Given the delayed kinetics of anti‐inflammatory cytokine responses relative to pro‐inflammatory mediators in acute‐phase immune reactions,^[^ ^34^, ^35^ ^]^ we extended observations to 96 and 120 h. This revealed that DC‐specific PFKFB2 knockout not only sustained suppression of pro‐inflammatory mediators but also significantly elevated IL‐10 levels at later timepoints. This anti‐inflammatory shift correlated with restored Treg/Th1 balance, indicating PFKFB2 knockout may reprogram DCs toward an immunoregulatory phenotype. Given IL‐10′s role in resolving lung injury,^[^ ^36^ ^]^ this mechanism likely contributes to the observed therapeutic efficacy.

Hypoxia is a fundamental pathological event in ALI. Minchenko et al.^[^ ^19^ ^]^ reported that hypoxia can promote the transcriptional expression of PFKFB2, possibly through the activation of HIF‐1α.^[^ ^13^ ^]^ As a central regulator of the hypoxic response, HIF‐1α has been shown to be upregulated in both PQ and LPS‐induced ALI models.^[^ ^20^, ^21^ ^]^ and to modulate LPS‐induced DC activation and function.^[^ ^23^ ^]^ In our study, PQ stimulation of BMDCs resulted in the upregulation of both HIF‐1α and PFKFB2, along with an increased proportion of mDCs. Treatment with the HIF‐1α inhibitor 2ME significantly reduced PFKFB2 transcription and decreased the proportion of mDCs, suggesting that PQ promotes PFKFB2 transcription via HIF‐1α activation. Although 2ME has been widely used as a HIF‐1α inhibitor in various disease models, including allergic airway inflammation,^[^ ^37^ ^]^ liver fibrosis,^[^ ^38^ ^]^ melanoma.^[^ ^39^ ^]^ and disc degeneration disease,^[^ ^40^ ^]^ it also exhibits non‐HIF‐related activities, such as estrogen receptor binding^[^ ^41^ ^]^ and disruption of microtubule networks.^[^ ^42^ ^]^ Critically, no current evidence links these non‐HIF mechanisms to PFKFB2 transcriptional regulation. This conclusion is further supported by our ChIP and EMSA experiments, which precisely mapped HIF‐1α binding to specific regions of the PFKFB2 promoter and demonstrated enhanced binding upon PQ stimulation. Nonetheless, to fully exclude off‐target contributions, future studies employing HIF‐1α knockdown via siRNA or alternative HIF‐1α inhibitors will be valuable. Collectively, these results indicate that PFKFB2 transcription is directly regulated by HIF‐1α. Activation of the HIF‐1α‐PFKFB2 axis likely promotes DC maturation by enhancing glycolysis and exacerbates inflammation in PQ/LPS‐induced ALI models, thereby highlighting a potential therapeutic target for ALI.

Pulmonary drug delivery systems have emerged as promising tools for treating lung diseases due to their rapid onset of action, high efficacy, and reduced adverse effects.^[^ ^43^, ^44^ ^]^ Nanotechnology, in particular, has shown significant potential in targeting DC activation and maturation, thereby enhancing drug delivery and therapeutic efficacy.^[^ ^45^, ^46^, ^47^ ^]^ For example, inhalation delivery systems targeting microRNA‐21 in lung macrophages have been shown to prevent fibrosis and restore lung function in COVID‐19‐induced ALI.^[^ ^48^ ^]^ Similarly, lipid nanoparticles designed to target liver macrophages have improved outcomes in acute liver injury.^[^ ^49^ ^]^ In this study, we employed a nanotechnology‐based strategy to target lung DC, inhibiting HIF‐1α to downregulate PFKFB2 expression, suppress DC maturation, and alleviate ALI. This approach enhances drug delivery and, concurrently, offers precise regulation of cellular signaling pathways, offering a molecular‐level strategy for ALI treatment. Furthermore, our findings in DC‐specific PFKFB2 knockout mouse models confirmed the therapeutic potential of PFKFB2 as a molecular target for ALI. Compared to previous studies, such as the nebulized core–shell lipid nanoparticle platform developed by Huang et al.^[^ ^27^ ^]^ our work represents a significant advancement in nanotechnology for targeting specific cell types in lung diseases.

PQ, a widely recognized oxidant, induces ALI through mechanisms involving oxidative stress and inflammatory cascades, similar to ALI caused by bacterial infections or sepsis. Using an LPS‐induced ALI mouse model, we verified that LPS also promotes DC maturation and exacerbates ALI via the HIF‐1α‐PFKFB2 signaling pathway. In human lung tissue, we observed significant upregulation of PFKFB2 expression, accompanied by increased DC maturation. These findings validate the conservation of PFKFB2 in regulating DC maturation across species and underscore its potential as a therapeutic target for clinical translation. Additionally, in the PQ‐induced ALI model, elevated mDCs proportion was accompanied by a decreased Treg/Th1 ratio, indicative of immune dysregulation. Targeted delivery of 2ME nanoparticles to DCs effectively reduced mDCs accumulation and restored Treg levels, thereby rebalancing the Treg/Th1 balance. In vitro mixed lymphocyte reactions further demonstrated that PQ‐primed BMDCs significantly increased the proportion of CD4⁺CD25⁺ T cells, a change that was reversed by 2ME treatment. These findings suggest that PQ promotes DC maturation, leading to Treg/Th1 imbalance and a disrupted local immune environment, potentially driving lung injury progression. Nonetheless, the precise relationship between DC maturation and T cell subset dynamics was not comprehensively addressed in this study. Future investigations will aim to elucidate the functional roles and interplay of distinct T cell subpopulations in PQ‐induced ALI, thereby refining our understanding of the immune microenvironment and informing targeted therapeutic strategies.

This study has several limitations. First, although our findings establish a key role for PFKFB2‐mediated glycolysis in driving DC maturation and ALI progression, they do not fully exclude contributions from other metabolic pathways, such as oxidative phosphorylation and fatty acid metabolism. Previous studies suggest that glycolysis may support de novo lipogenesis to meet biosynthetic demands during DC activation,^[^ ^50^ ^]^ indicating potential crosstalk between pathways. While PFKFB2 is a known glycolytic regulator, its direct involvement in non‐glycolytic metabolism remains unclear. Future studies using metabolic flux analysis and targeted inhibitors will be essential to delineate the broader metabolic networks involved in DC‐mediated inflammation. Second, while BMDCs are widely used to study immunometabolic mechanisms and have demonstrated good consistency with in vivo findings across various disease models,^[^ ^51^, ^52^, ^53^ ^]^ they may not fully recapitulate the complex metabolic landscape of DCs within the ALI microenvironment. To address this limitation, we conducted additional in vivo experiments. After validating the role of PFKFB2 in BMDCs in vitro, we further performed targeted modulation of DCs in vivo, which strongly supported the promotive role of PFKFB2 in DC activation and maturation during ALI. These in vivo results complement the in vitro findings and help to partially offset the limitations associated with the in vitro model.

In conclusion, this study identifies PFKFB2 as a critical regulator of DC maturation and a key driver of ALI progression. The HIF‐1α‐PFKFB2 signaling pathway promotes DC maturation and amplifies inflammatory responses, thereby exacerbating ALI. Preliminary evidence from human lung tissues indicates that the HIF‐1α‐PFKFB2 axis may be conserved in ALI of diverse etiologies. Targeting this axis may provide a potential therapeutic avenue for further exploration in the treatment of ALI, offering new avenues for precision medicine and clinical intervention.

## 4 Experimental Section

### Human Samples

Lung tissue samples were collected from four patients with lung transplantation due to PQ poisoning, four patients with 2°BP^[^ ^54^ ^]^ through pathological biopsy, and one patient with COVID‐19 pneumonia who underwent lung transplantation. As controls, lung tissues were obtained from the resection margins of three patients undergoing tumor removal surgery for lung cancer. Immunofluorescence staining was performed on all collected samples to assess relevant molecular markers. The study protocol, including the use of human lung tissue samples, was approved by the Ethics Committee of The First Affiliated Hospital of Zhengzhou University (Approval Number: 2024‐KY‐1654‐001).

### Mice

C57BL/6*J* mice were obtained from Beijing Vital River Laboratory Animal Technology Co., Ltd. CD11c‐Cre mice (stock: C01063) and PFKFB2^fl/fl^ (stock:S‐CKO‐04248) mice were obtained from Cyagen Biosciences Inc. The DC specific PFKFB2‐null mice were generated by breeding PFKFB2^fl/fl^ mice with CD11c‐Cre transgenic mice (PFKFB2^ΔCD11c^). All experimental mice used were males and were housed in a standard laboratory environment and allowed to have a rodent animal diet and water at will. Ethical approval for all animal experiments was obtained from the Animal Ethics Committee of Zhengzhou University (Permit Number: ZZU‐LAC20230915[06]).

### Animal Experiment

(Wild‐type mouse experiments) C57BL/6J mice aged 6–8 weeks were randomly assigned to experimental and control groups. For the induction of lung injury, mice were exposed to either an intraperitoneal injection of PQ (35 mg kg^−1^, 36 541, Sigma–Aldrich, St. Louis, MO, USA) or an intratracheal administration of LPS (5 mg kg^−1^, L2880, Sigma–Aldrich, St. Louis, MO, USA). The treatment group received daily intratracheal administrations of 2ME NPs (20 mg kg^−1^), while the control group received an equivalent volume of Ctrl NPs administered via the same route. (PFKFB2 conditional knockout mouse experiments) PFKFB2^fl/fl^ and PFKFB2^ΔCD11c^ mice, aged 8–10 weeks, were randomly assigned to experimental and control groups. For the induction of lung injury, mice were exposed to PQ (35 mg kg^−1^, intraperitoneally), LPS (5 mg kg^−1^, intratracheally). In the survival experiments, PQ was administered at a higher dose of 40 mg kg^−1^. Control mice received PBS via the same route as the experimental treatment (intraperitoneally or intratracheally) to ensure consistency. After 72 h of PQ treatment^[^ ^55^ ^]^ or 24 h of LPS treatment,^[^ ^56^ ^]^ mice were euthanized, and blood and lung tissues were harvested for further analyses. In the survival experiment, mice were monitored every 12 h for 15 days after drug administration to assess survival status. Survival data were analyzed using the Log‐rank test.

### Hematoxylin and Eosin (H&E) Staining of Lung Tissue

For histopathological evaluation, lung lobes were fixed in formalin, thoroughly washed, dehydrated, and embedded in heated paraffin wax. The embedded tissues were sectioned into 4‐µm‐thick slices and subsequently stained with hematoxylin and eosin to assess pathological changes. The stained slides were scanned using the Pannoramic MIDI system (3DHISTECH, Budapest, Hungary). A semi‐quantitative lung injury score was assigned to each sample based on the criteria established by Gustavo Matute‐Bello et al.^[^ ^57^ ^]^ This scoring system evaluates parameters such as neutrophil infiltration or aggregation in the airspace or vessel wall, alveolar wall thickening, and the presence of hyaline membranes and proteinaceous debris in the alveoli. Ten randomly selected fields per sample were analyzed by an observer blinded to the experimental groups to ensure objectivity and reliability in the assessment.

### Mouse Lung Single Cell Suspension Preparations

The lung tissues were then minced using ophthalmic scissors and digested in RPMI 1640 medium containing 100 µL of collagenase D (1 mg mL^−1^), 5 µL of DNase I (0.03 mg mL^−1^), and 10 µL of CaCl₂ (5 mm) (11088866001, 10104159001, C5670, Sigma–Aldrich, St. Louis, MO, USA) in a total volume of 1 mL. The digestion mixture was incubated at 37 °C with shaking at 300 rpm for 1 h to ensure enzymatic dissociation of the tissue. Following digestion, the mixture was filtered through a 70‐µm nylon mesh to remove undigested fragments. The resulting filtrate was treated with 1 mL of stop buffer and gently pipetted to achieve a homogeneous suspension before being centrifuged at 300 g for 5 min. Red blood cells were lysed using ACK Lysis Buffer (CS0001, LeiGene Biotech Co., Ltd., Beijing, China) by incubating the suspension at room temperature for 2 min, followed by neutralization with PBS. This procedure yielded a purified single‐cell suspension suitable for downstream analyses.

### Flow Cytometry

For analysis of surface markers, cells were first stained in PBS containing 2% (wt/vol) fetal bovine serum (FBS) for 30 min on ice. To assess cell viability and minimize nonspecific binding, cells were initially incubated with a fixable viability dye (Zombie Aqua Fixable Viability Kit, 423102, BioLegend, San Diego, USA) and an anti‐CD16/32 antibody (101319, BioLegend, San Diego, USA) for 10 min at room temperature, this step was followed by surface marker staining. For intracellular staining, cells were fixed with the Cyto‐Fast Fix/Perm Buffer Set (426803, BioLegend, San Diego, USA) according to the manufacturer's instructions. For intracellular staining of transcription factors, cells were fixed with the True‐Nuclear Transcription Factor Buffer Set (424401, BioLegend, San Diego, USA) according to the manufacturer's instructions. The following antibodies were used: anti‐CD11c (117308), anti‐CD40 (124612), anti‐CD80 (104714), anti‐CD86 (105012), anti‐CD45 (103115), anti‐CD64 (139313), anti‐MerTK (151510), anti‐MHC‐II (107639), anti‐CD80 (104705), anti‐CD86 (105041), anti‐CD3 (100203), anti‐CD4 (116012), anti‐FOXP3 (126403), anti‐T‐bet (644823), anti‐CD25 (102011), anti‐CD11b (101225), anti‐F4/80 (123121) (all from BioLegend, San Diego, USA); anti‐PFKFB2 (sc‐377416, Santa Cruz, CA, USA). Flow cytometry data were acquired using FACSARIA III (BD Biosciences, Franklin Lakes, NJ, USA) or ACEA NOVOCYte3130 (Agilent Technologies, Inc., Santa Clara, CA, USA) and analyzed with FlowJo software (Version 10.1, Tree Star, Ashland, Oregon, USA). As described previously,^[^ ^58^, ^59^ ^]^ all gating strategies are shown in Figure S13 (Supporting Information).

### BMDCs Culture and Treatment

BMDCs were cultured and treated to investigate their response to specific stimuli. Femurs and tibiae were harvested from 6–8‐week‐old male C57BL/6J mice, and bone marrow cells were flushed out using RPMI 1640 medium. After lysing red blood cells, the cell suspension was filtered through a 70‐µm nylon mesh to remove bone fragments and debris. Bone marrow cells were seeded at a density of 3 × 10^6^ cells per well in 6‐well plates containing 2 mL of RPMI 1640 medium (Thermo Fisher Scientific Inc., Waltham, Massachusetts, USA) supplemented with 10% heat‐inactivated FBS, 55 mm 2‐mercaptoethanol (Invitrogen, Carlsbad, California, USA), 1% penicillin‐streptomycin, recombinant mouse GM‐CSF (50 ng mL^−1^, 315‐03, PeproTech, Cranbury, New Jersey, USA), and IL‐4 (20 ng mL^−1^, 214‐14, PeproTech, Cranbury, New Jersey, USA). To maintain optimal culture conditions, 70% of the medium was replaced with fresh medium containing GM‐CSF and IL‐4 on day 3. By day 6, semi‐suspended and loosely adherent cells were collected via gentle PBS washing and used as immature BMDCs for subsequent experiments (Figure S1a–d, Supporting Information). These BMDCs were stimulated with 200 µm PQ and treated with either 0.5 µm 2ME or 20 µm 2‐DG (S1233, S4701, Selleck Chemicals, Houston, TX, USA) for 24 h.

### Seahorse XF Cell Glycolysis Stress Test Assay

To evaluate the glycolytic capacity of BMDCs, the Seahorse Glycolysis Stress Test Kit (103020–100, Agilent Technologies, Santa Clara, California, USA) was utilized. BMDCs were seeded at a density of 70 000 cells per well onto Cell‐Tak‐coated XF96 cell culture microplates (103794‐100, Agilent Technologies, Santa Clara, California, USA) in Seahorse XF RPMI Medium (103576‐100, Agilent Technologies, Santa Clara, California, USA) supplemented with 2 mm glutamine, following the manufacturer's protocol. The assay involved sequential injections of specific compounds into designated ports of the Seahorse analyzer: Port A contained glucose (final concentration 10 mm) to initiate glycolysis, Port B contained oligomycin (final concentration 1 µm) to inhibit ATP synthesis and reveal the glycolytic reserve, and Port C contained 2‐DG (final concentration 50 mm) to inhibit glycolysis and confirm the specificity of glycolytic measurements. The assay was conducted using an Agilent Seahorse XFe96 analyzer, and data were analyzed with Wave Desktop and Controller Software (version 2.6.1, Agilent Technologies, Santa Clara, California, USA). This comprehensive approach provided detailed insights into the glycolytic profiles of BMDCs under various experimental conditions.

### Dual Luciferase Reporter Gene Assay

GV712‐HIF1α and GV712‐empty expression plasmids, along with WT or MUT PFKFB2 promoter‐luciferase reporter constructs, were designed and provided by Shanghai Genechem Co., Ltd. The 293T cells were seeded in 96‐well plates and co‐transfected with the GV712‐HIF1α or GV712‐empty plasmids, together with either WT or MUT PFKFB2 promoter‐luciferase reporter constructs, using PEI transfection reagent. After 48 h of incubation, luciferase activity was measured using the Duo‐Lite Luciferase Assay System (DD1205‐01, Vazyme Biotech Co., Ltd., Nanjing, China). Firefly luciferase activity was normalized to Renilla luciferase activity. All experiments were performed in triplicate.

### Electrophoretic Mobility Shift Assay (EMSA)

Nuclear extracts were prepared using the Nuclear Protein Extraction Kit (P0027, Beyotime, Shanghai, China), and protein concentrations were measured using the BCA Protein Assay Kit (A55865, Thermo Fisher Scientific, Waltham, MA, USA). Labeled and unlabeled probes (Sangon Biotech Co., Ltd., Shanghai, China) containing the binding site were synthesized according to the sequences provided in Table S3 (Supporting Information). DNA binding activity of HIF‐1α was determined using a chemiluminescent EMSA Kit (GS009, Beyotime, Shanghai, China).

### Chromatin Immunoprecipitation (ChIP)

ChIP assay was performed according to the manufacturer's instructions of the EpiQuik Plant ChIP kit (p‐2002‐2, Epigentek Group Inc., Farmingdale, New York, USA). Cells were cross‐linked with formaldehyde and then collected for protein extraction. The chromatin was sheared to 100–1,000 bp by sonication and immunoprecipitated with anti‐HIF‐1α antibody (ab228649, Abcam, Cambridge, UK). An equivalent amount of non‐specific IgG was used as a control. The immunoprecipitated protein‐DNA complex was purified and then captured with protein‐G beads. Relative enrichment values were calculated by dividing the immunoprecipitated DNA by the input DNA. The primer sequences for PFKFB2 are provided in Table S3 (Supporting Information).

### Statistical Analysis

Statistical analyses were conducted using SPSS software (version 26.0, SPSS Inc., Chicago, IL, USA). Biological replicates were included to account for variability, and sample sizes were determined through statistical power analysis. Before applying parametric tests, data normality was assessed using the Shapiro‐Wilk test, and homogeneity of variances was evaluated using Levene's test. Normally distributed data were presented as mean ± standard deviation (SD). For two‐group comparisons, two‐tailed Student's *t*‐tests were performed, while one‐way ANOVA followed by Fisher's LSD post hoc analysis was used for comparisons among multiple groups. Non‐normally distributed data were described by the median and interquartile range [M(p25, p75)]. Comparison between two groups was conducted by the Mann‐Whitney U test. Kaplan‐Meier survival analysis was conducted using the Log‐rank test to evaluate survival outcomes. *p* < 0.05 was considered statistically significant.

## Conflict of Interest

The authors declare no conflict of interest.

## Supporting information



Supporting Information

## Data Availability

The data that support the findings of this study are available on request from the corresponding author. The data are not publicly available due to privacy or ethical restrictions.
