# Supplementary material for: PFKFB2‐Driven Glycolysis Promotes Dendritic Cell Maturation and Exacerbates Acute Lung Injury
Source: Adv Sci (Weinh). 2025 Jul 30;12(37):e02428. doi: 10.1002/advs.202502428 (PMC12499423; doi:10.1002/advs.202502428)
Supplement: Supplementary file 1 — Supporting Information [file ADVS-12-e02428-s001.docx]

**PFKFB2-Driven Glycolysis Promotes Dendritic Cells Maturation and Exacerbates Acute Lung Injury**

*Ding Yuan^a1^, Fang Yang^a1^, Linlin Hou^a1^, Yan Zhang^a^, Xin Pang^b^, Yuqi Du^a^, Hongyi Yan^a^, Huanzhou Zhu^a^, Yue Cheng^a^, Yue Wu^a^, Pinpin Jiang^a^, Mengnan Guo^a^, Mengying Zhang^c^, Jinjie Guo^a^, Huihui Hao^a^, Yong Jiang^c*^, Yi Li^d*^, Yanxia Gao^a*^*

**Supplemental Experimental Procedures**

Bone marrow-derived macrophages (BMDMs) culture: Femurs and tibiae were harvested from 6–8-week-old male C57BL/6J mice, and bone marrow cells were flushed out using DMEM medium. After lysing red blood cells, the cell suspension was filtered through a 70-µm nylon mesh to remove bone fragments and debris. Bone marrow cells were seeded at a density of 3×10^6^ cells per well in 6-well plates containing 2 mL of DMEM medium (Thermo Fisher Scientific Inc., Waltham, Massachusetts, USA) supplemented with 10% heat-inactivated FBS, penicillin, streptomycin, macrophage colony-stimulating factor (M-CSF, 20ng/ml; Beyotime Biotechnology, Shanghai, China). The medium was refreshed on day 3, and adherent cells were collected on day 6 for subsequent experiments.

Pulmonary delivery of intratracheal administration: To facilitate intratracheal administration, mice were anesthetized using ether, and their upper incisors were secured to an operating platform to ensure stability during the procedure. A cold light source was employed to illuminate the cervical region, enhancing visibility, while the tongue was gently extended using tweezers to expose the vocal cords. Using a solution-based pulmonary nebulizer (TOW Intelligent Technology Co., Ltd., Shanghai, China), 50 μL of either 2-methoxyestradiol@DSPE-PEG-MAN nanoparticles (2ME NPs) solution or LPS solution was administered intratracheally to the experimental group. The control group received an equivalent volume of either nanoparticles without 2ME loading (Ctrl NPs) solution or PBS, depending on the experimental condition, administered using the same procedure.

Immunohistochemistry: To investigate the expression of myeloperoxidase (MPO) in lung tissue, immunohistochemistry was performed. Lung sections underwent antigen retrieval in citric acid buffer, followed by heating in a 100°C water bath for 15 min. After cooling, endogenous peroxidase activity was blocked by incubating the sections with 3% hydrogen peroxide for 25 min. To prevent nonspecific binding, sections were treated with 3% bovine serum albumin (BSA) for 30 min. The sections were then incubated overnight at 4°C with a primary antibody specific to MPO (1:300 dilution, [ab208670](https://www.abcam.cn/products/primary-antibodies/myeloperoxidase-antibody-epr20257-ab208670.html), Abcam, Cambridge, UK). The next day, the sections were washed with PBS and incubated with a biotinylated goat anti-mouse secondary antibody (1:500 dilution, G1213, Servicebio, Wuhan, China) at room temperature for 60 min, followed by streptavidin-horseradish peroxidase incubation at 37°C for 10 min. Visualization of MPO expression was achieved using diaminobenzidine (DAB) staining, after which the sections were subjected to routine dehydration, transparency, and sealing with neutral resin. Images of each section were collected under Pannoramic MIDI (3DHISTECH, Budapest, Hungary). Aipathwell (Servicebio, Wuhan, China) was used to scan the slides and quantify MPO levels in the lung tissue (Positive cell ratio = Number of MPO-positive cells / total number of cells).

Immunofluorescence: To investigate protein expression and cellular localization in human lung tissue samples, immunofluorescence analysis was performed. The samples were fixed in paraformaldehyde overnight and subsequently dehydrated in a sucrose solution. Coronal frozen sections, each 20 μm in thickness, were prepared using a cryostat (Leica Biosystems, Wetzlar, Germany). Antigen retrieval was conducted using EDTA buffer (pH 8.0, G1206, Servicebio, Wuhan, China) with microwave treatment. Following this, the sections were permeabilized with 0.3% Triton X-100 (T8200, Solarbio, Wuhan, China) and blocked with 10% BSA to minimize nonspecific binding. Primary antibodies, including CD11c (1:500 dilution, sc-81853, Santa Cruz, CA, USA), PFKFB2 (1:300 dilution, sc-377416, Santa Cruz, CA, USA), and HLA-DR (1:400 dilution, ab92511, Abcam, Cambridge, UK), were applied, and the sections were incubated overnight at 4°C. The next day, the sections were incubated with secondary antibodies at room temperature for 2 h in the dark to preserve fluorescence integrity. Nuclei were counterstained with 4',6-diamidino-2-phenylindole (DAPI, S2110, Solarbio, Wuhan, China) to facilitate cellular visualization. Fluorescence images were captured using a fluorescence microscope (NIKON Eclipse ci), and quantitative analysis was conducted using ImageJ software (version 1.52o, NIH, Bethesda, MD, USA). Parameters such as the number of positive cells were quantified to assess expression levels and spatial distribution.

Preparation of 2ME NPs: The 2ME NPs were prepared using the thin-film hydration method. Briefly, 100 mg of DSPE-PEG-MAN (mannose-modified PEG-DSPE, R-H54510, Xi’an Rui Xi Biotechnology Co., Ltd., China) and 10 mg of 2ME (S1233, Selleck Chemicals LLC, Houston, Texas, USA) were mixed at a molar ratio of 10:1 in a round-bottom flask. The mixture was dissolved in 10 mL of chloroform under gentle agitation to ensure complete dissolution. The solvent was then evaporated under reduced pressure using a rotary evaporator, forming a thin, uniform lipid film on the inner surface of the flask. To eliminate residual chloroform, the flask containing the lipid film was placed in a vacuum oven and dried overnight. Subsequently, 10 mL of saline was added to hydrate the film, followed by vigorous vortexing for 3 min. The hydrated mixture was further processed by water bath sonication at 40°C for 15 min to facilitate nanoparticle formation. To achieve a homogeneous nanoparticle suspension with reduced particle size, the solution was probe-sonicated for 1 minute at 20 W using a titanium probe, yielding an opalescent suspension. The resulting nanoparticle suspension was sequentially filtered through 0.45 μm and 0.22 μm microporous membranes to remove unencapsulated drug aggregates and ensure uniform nanoparticle size distribution. The purified nanoparticles were stored in airtight containers at 4°C until further use. As a control, Ctrl NPs were prepared following the same protocol.

Characterization of 2ME NPs: Characterization of the 2ME NPs was conducted using multiple analytical techniques to ensure their quality and uniformity. Particle size and zeta potential were analyzed using a Malvern particle size analyzer. For particle size measurements, a suitable amount of the nanoparticle aqueous dispersion was subjected to ultrasonic treatment to achieve uniform distribution, with each sample measured in triplicate. Similarly, for zeta potential analysis, the nanoparticle aqueous dispersion was ultrasonicated for uniform distribution, and 800 μL of the sample was transferred to a zeta potential sample cell for measurement. Transmission electron microscopy (TEM) was employed to observe the morphology and confirm the spherical structure of the nanoparticles. A 10 μL aliquot of the nanoparticle aqueous dispersion was ultrasonicated for uniform distribution, dropped onto a 200-mesh copper grid, and dried at room temperature before imaging under the TEM. Additionally, UV-visible absorption spectroscopy was performed by dispersing the nanoparticle aqueous dispersion uniformly using ultrasonication, and the absorption spectrum was recorded using a UV-3600 UV-Vis spectrophotometer. Changes in absorption peaks were analyzed to verify the successful preparation of 2ME NPs and to assess potential structural interactions between 2ME and the lipid matrix. The drug carrier in our study, DSPE-PEG-MAN, is a phospholipid derivative. It is well known that phospholipases are widely distributed in biological systems. Following cellular uptake (e.g., by dendritic cells), nanoparticles accumulate in lysosomes, where they encounter high concentrations of phospholipases. These enzymes rapidly degrade the lipid matrix of nanoparticles, triggering destabilization and subsequent drug release for therapeutic activity. To characterize this process, we employed the well-established dialysis bag method. The results showed that more than 90% 2ME was liberated in the presence of phospholipase after 24 h incubation, while the nanoparticles were stable with only 30% 2ME release in phospholipase-free media. This phospholipase-responsive behavior suggests that the nanoparticles maintain structural integrity under delivery conditions (e.g., pulmonary mucus, where phospholipase activity is low) while undergoing rapid biodegradation and drug release upon lysosomal uptake by DCs—a mechanism that optimizes both therapeutic delivery and biosafety. To assess the colloidal stability of the prepared nanoparticles, the nanoparticle dispersion was stored at 4°C and systematically characterized over a 7-day observation period. Daily measurements of hydrodynamic diameter and zeta potential were performed using a Dynamic Light Scattering and Zeta Potential measurement system (Nano ZS90, Malvern Instruments, UK). As illustrated in Figure S7b,c, Supporting Information, the nanoparticles demonstrated excellent stability throughout the evaluation period, with no significant alterations in either particle size or surface charge. This remarkable stability profile, evidenced by minimal size variation (<5%) and consistent zeta potential values (±2 mV), strongly supports the formulation's potential for clinical translation and further pharmaceutical development.

Glucose uptake assay: To evaluate glucose uptake in bone marrow-derived dendritic cells (BMDCs), cells were first incubated in glucose-free RPMI 1640 medium supplemented with 10% FBS for 30 min to deplete intracellular glucose reserves. Following this pre-incubation, 2-NBDG (N13195, Thermo Fisher Scientific Inc., Waltham, Massachusetts, USA), a fluorescent glucose analog, was added to the medium at a final concentration of 100 μM and incubated for 15 min to allow cellular uptake. After incubation, cells were collected and washed twice with PBS to remove excess 2-NBDG. Glucose uptake was subsequently analyzed using flow cytometry, with data processing performed using FlowJo software. This assay provided quantitative insights into the glucose uptake capacity of BMDCs under specific experimental conditions.

RNA Interference: The siRNA oligonucleotides targeting PFKFB2 were designed and synthesized by RiboBio Co., Ltd. (Guangzhou, China). On day 6 of BMDCs culture, cells (1×10^6^ cells/well) were seeded into 6-well plates and transfected with PFKFB2 siRNA or scrambled siRNA using Lipofectamine™ RNAiMAX (13778075, Thermo Fisher Scientific Inc., Waltham, Massachusetts, USA) according to the manufacturer’s protocol. Post-transfection, gene silencing efficiency was confirmed through Western blot analysis. The siRNA sequence utilized in this study was 5'-GGAGATCCAGGACCTTAAA-3'.

Ex vivo metabolite analysis: To profile the metabolic alterations induced by PQ, BMDCs were treated with or without PQ (200 μM) for 24 h. Metabolites were extracted using ice-cold methanol to ensure the preservation of endogenous metabolite integrity. The extracted metabolites were analyzed using liquid chromatography-mass spectrometry (LC-MS), providing a comprehensive overview of the metabolic landscape. Data acquisition was conducted using a 5500 QTRAP mass spectrometer (AB SCIEX, Foster City, California, USA), and subsequent analysis was performed using Multi-Quant software (Applied Biosystems/Sciex). Normalization of metabolite levels was carried out using internal standards, and statistical analyses, were employed to identify significant metabolic changes between the groups.

Enzyme-linked immunosorbent assay (ELISA): To quantify cytokine levels in the culture supernatants, ELISA kits were utilized to measure IL-10, IL-1β, IL-6, and TNF-α (E-EL-M0046, E-EL-M0037, E-EL-M0044, E-EL-M3063, Elabscience, Wuhan, China). The assay was conducted following the manufacturer’s instructions, with blank control wells and standard wells included to ensure accuracy and reliability. All measurements were performed in triplicate to minimize variability and enhance the robustness of the data. Absorbance was measured at 450 nm using a microplate reader, and a standard curve was generated to calculate the concentrations of cytokines in the test samples.

Western blot: Protein extracts were separated by SDS-PAGE and subsequently transferred onto PVDF membranes. To minimize nonspecific binding, membranes were blocked with 5% fat-free milk and incubated overnight at 4°C with primary antibodies targeting PFKFB2 (1:5000, ab241506, Abcam, Cambridge, UK), HIF-1α (1:1000, ab179483, Abcam, Cambridge, UK), and β-actin (1:5000, ab6276, Abcam, Cambridge, UK). Following this, membranes were incubated with secondary antibodies for 2h at room temperature. Protein bands were visualized using enhanced chemiluminescence (32106, Thermo Fisher Scientific Inc., Waltham, Massachusetts, USA) and imaged with an Amersham Imager 600 (GE Healthcare, CA, USA). Quantitative analysis of the protein bands was performed using ImageJ software, with normalization conducted against β-actin to account for variations in protein loading.

Quantitative Real-Time PCR (qRT-PCR): To assess gene expression levels, total RNA was extracted using Trizol reagent (15596026CN, Thermo Fisher Scientific Inc., Waltham, Massachusetts, USA). Complementary DNA (cDNA) was synthesized from the extracted RNA using the TransScript kit (RR047A, Takara Bio Inc., Kusatsu, Shiga, Japan). qRT-PCR was performed using SYBR Green (RR802A, Takara Bio Inc., Kusatsu, Shiga, Japan) on a QuantStudio5 real-time PCR system (Thermo Fisher Scientific Inc., Waltham, Massachusetts, USA). β-actin served as the internal control to normalize gene expression levels. All experiments were conducted in triplicate to ensure reproducibility. Primer sequences used for qRT-PCR are provided in Table S3.


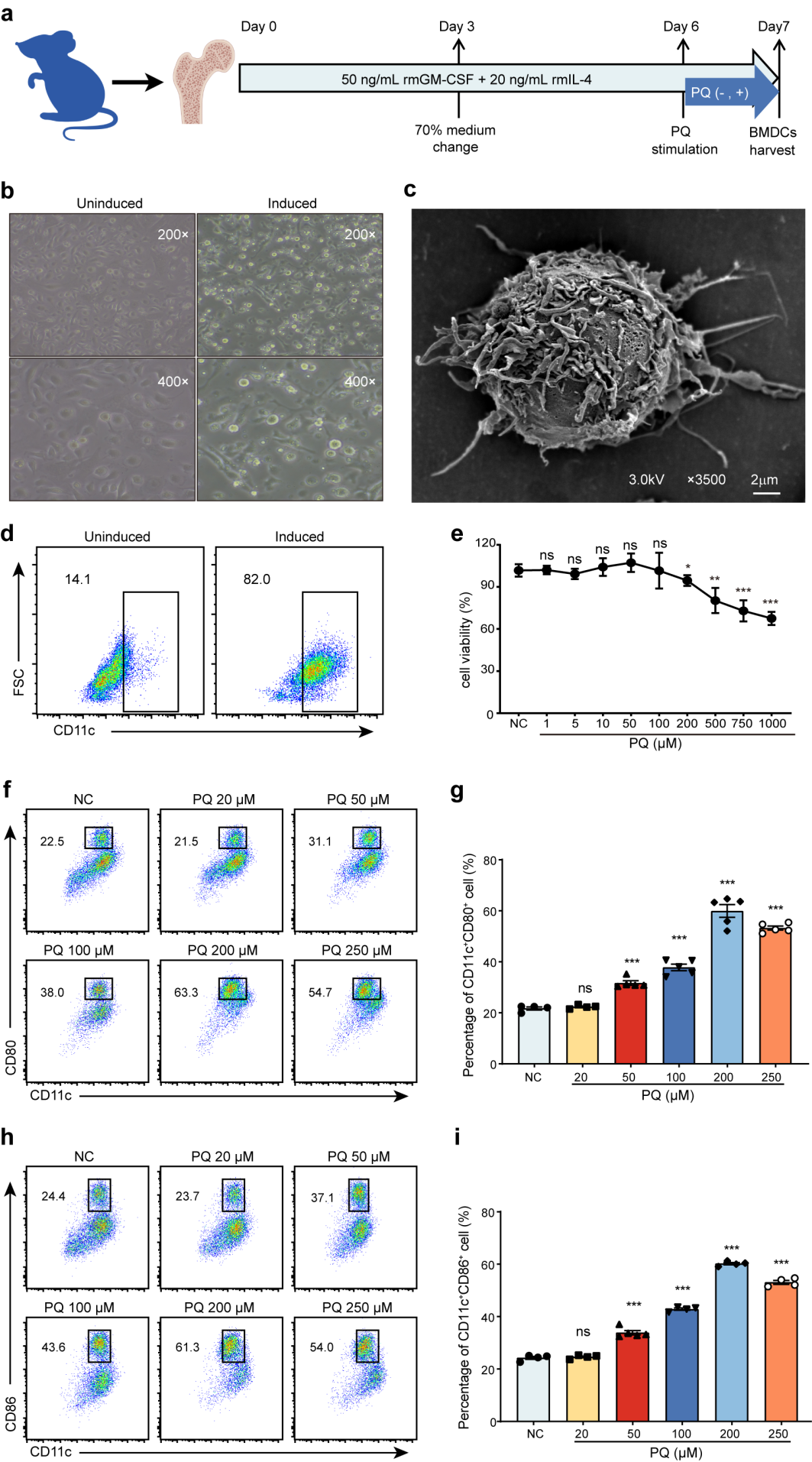


**Figure S1.** Identification of BMDCs and the effect of PQ on BMDCs viability and maturation. a) The extraction and induction culture process of BMDCs. b,c) Light microscopy and electron microscopy images of in vitro cultured BMDCs. d) Flow cytometry analysis of CD11c expression on bone marrow cells with or without GM-CSF and IL-4 induction. e) Cell viability in each group after 24h of treatment with different concentrations of PQ (0, 1, 5, 10, 50, 100, 200, 500, 750, 1000 μM) (n=4 wells/group). f,g) Flow cytometry analysis of CD80 expression on BMDCs after 24h of treatment with different concentrations of PQ (0, 20, 50, 100, 200, 250 μM) (n=5 wells/group). h,i) Flow cytometry analysis of CD86 expression on BMDCs after 24h of treatment with different concentrations of PQ (0, 20, 50, 100, 200, 250 μM) (n=4-5 wells/group). *^ns^P*>0.05, *^*^P*<0.05, *^**^P*<0.01, *^***^P*<0.001. All values are means ± SD, and significance was determined by one-way analysis of variance (ANOVA) with Fisher’s LSD post hoc analysis.


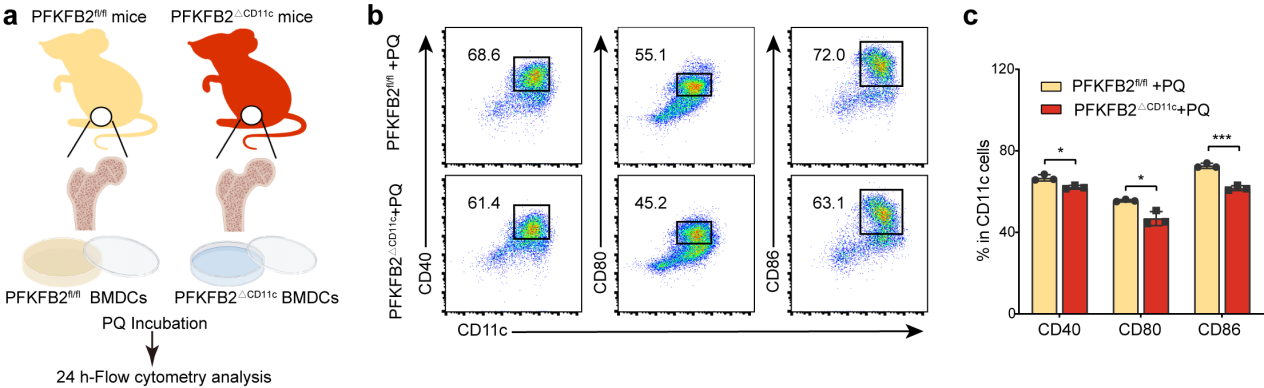


**Figure S2.** PFKFB2 deficiency impairs PQ induced BMDCs maturation. a) Experimental design created with figdraw.com. b,c) Flow cytometry detection of the maturation proportion of BMDCs from PFKFB2^fl/fl^ and PFKFB2^ΔCD11c^ mice treated with 200 μM PQ for 24h (n=3 wells/group). *^*^P*<0.05, *^***^P*<0.001. All values are means  ± SD, and significance was determined by two-tailed Student’s t-test.


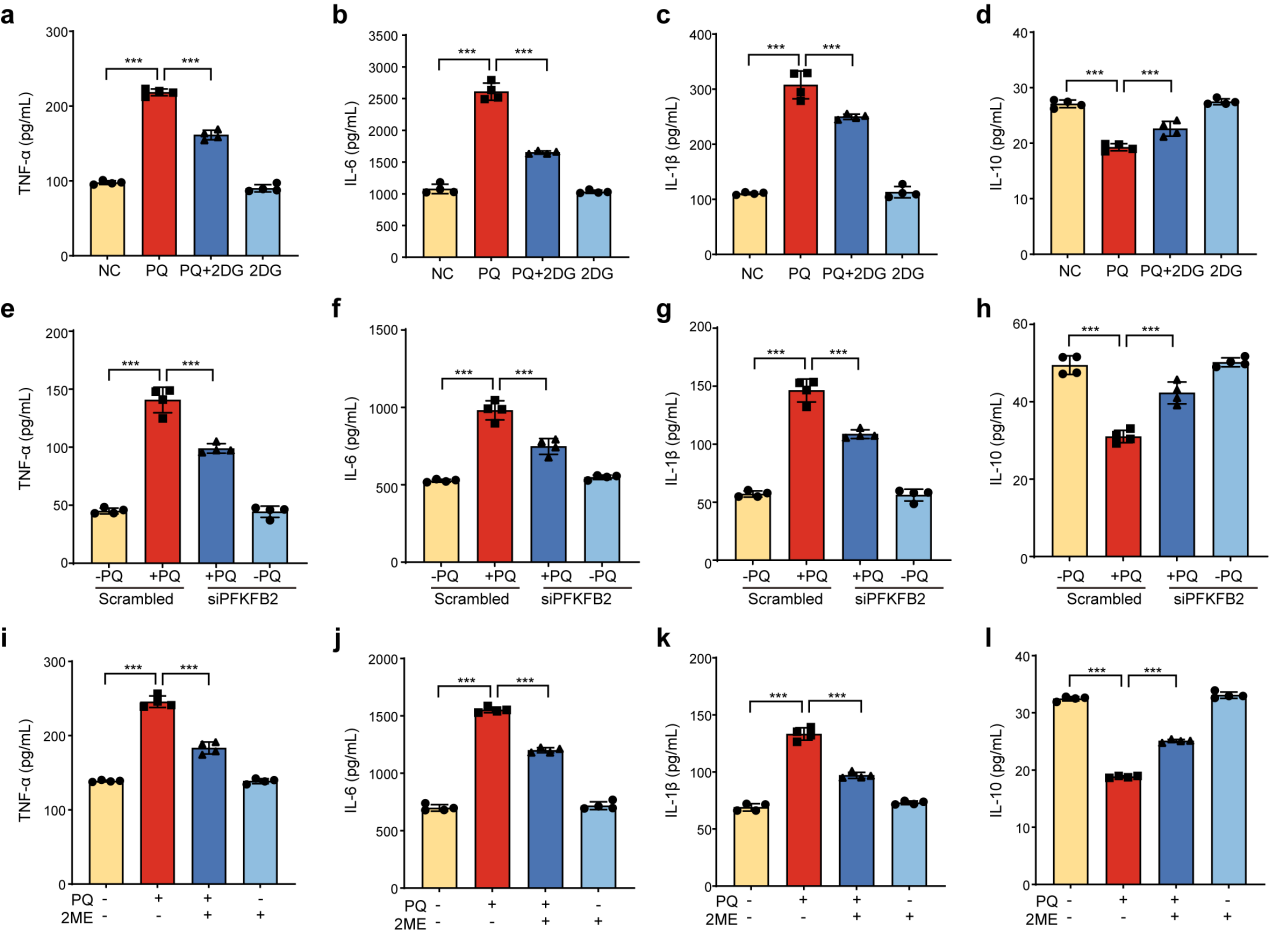


**Figure S3.** ELISA detection of TNF-α, IL-6, IL-1β, and IL-10 secretion in BMDCs a under different conditions. a-d) Levels of TNF-α, IL-6, IL-1β and IL-10 in BMDCs treated with 20 μM 2-DG and 200 μM PQ for 24h (n=4 wells/group). e-h) Levels of TNF-α, IL-6, IL-1β and IL-10 in BMDCs with or without PFKFB2 knockdown treated with 200 μM PQ for 24h (n=4 wells/group). i-l) Levels of TNF-α, IL-6, IL-1β and IL-10 in BMDCs treated with 200 μM PQ and 2 μM 2ME for 24h (n=4 wells/group). *^***^P*<0.001. All values are means ± SD, and significance was determined by one-way analysis of variance (ANOVA) with Fisher’s LSD post hoc analysis.


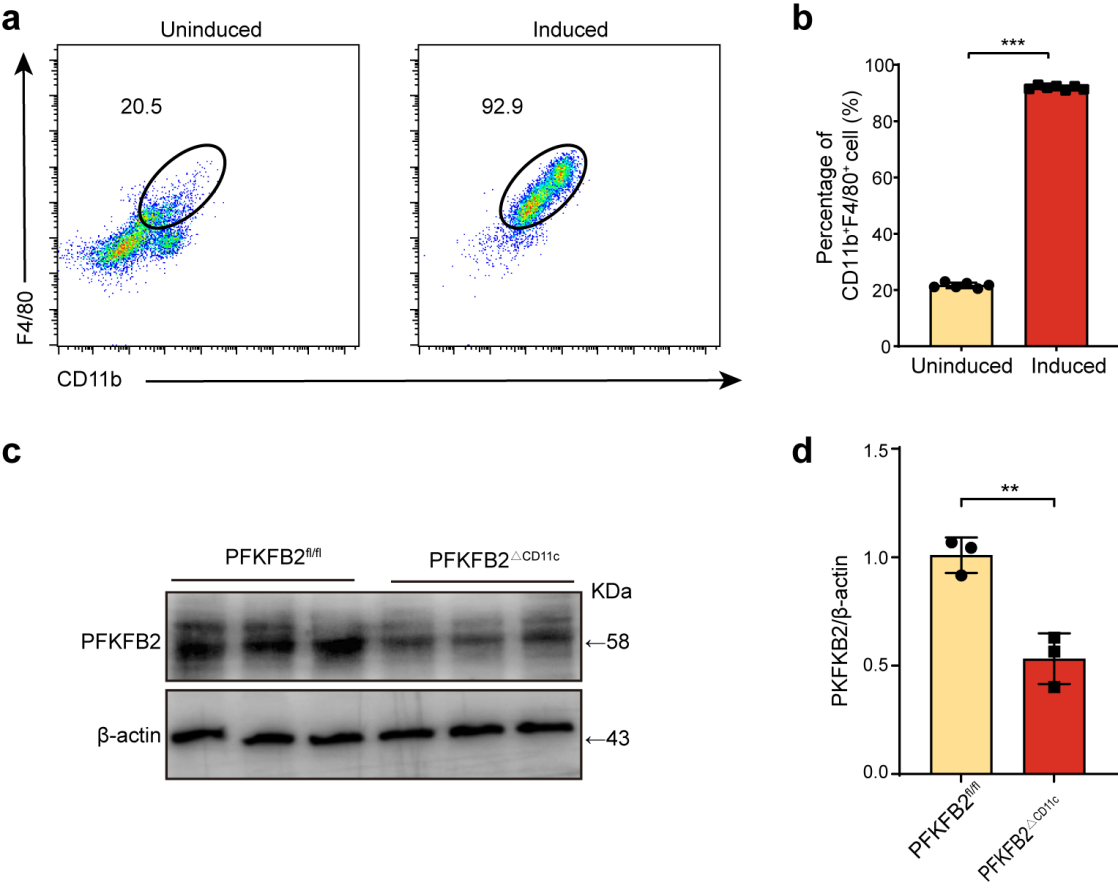


**Figure S4.** In vitro culture and characterization of BMDMs. a,b) Flow cytometry analysis of CD11b and F4/80 expression on bone marrow cells with or without M-CSF induction and flow cytometry quantification (n=6-7 wells/group). c,d) Western blot detection of PFKFB2 protein levels in BMDMs from WT and PFKFB2^ΔCD11c^ mice and quantification (n=3 samples/group). *^**^P*<0.01, *^***^P*<0.001. All values are means  ± SD, and significance was determined by two-tailed Student’s t-test.


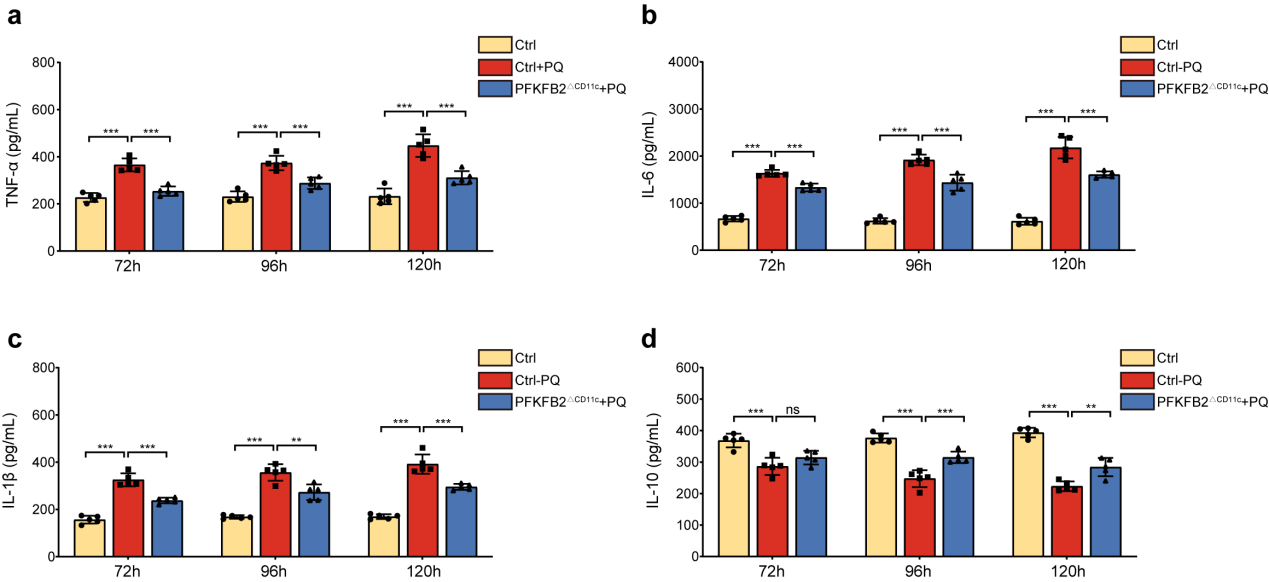


**Figure S5.** PFKFB2 knockout in DCs mitigates PQ-ALI by inhibiting DCs maturation. a-d) serum cytokine levels in mice at 72h, 96h and 120h after PQ treatment measured by ELISA (n=5 mice/group). *^ns^P*>0.05, *^**^P*<0.01, *^***^P*<0.001. All values are means ± SD, and significance was determined by one-way analysis of variance (ANOVA) with Fisher’s LSD post hoc analysis.


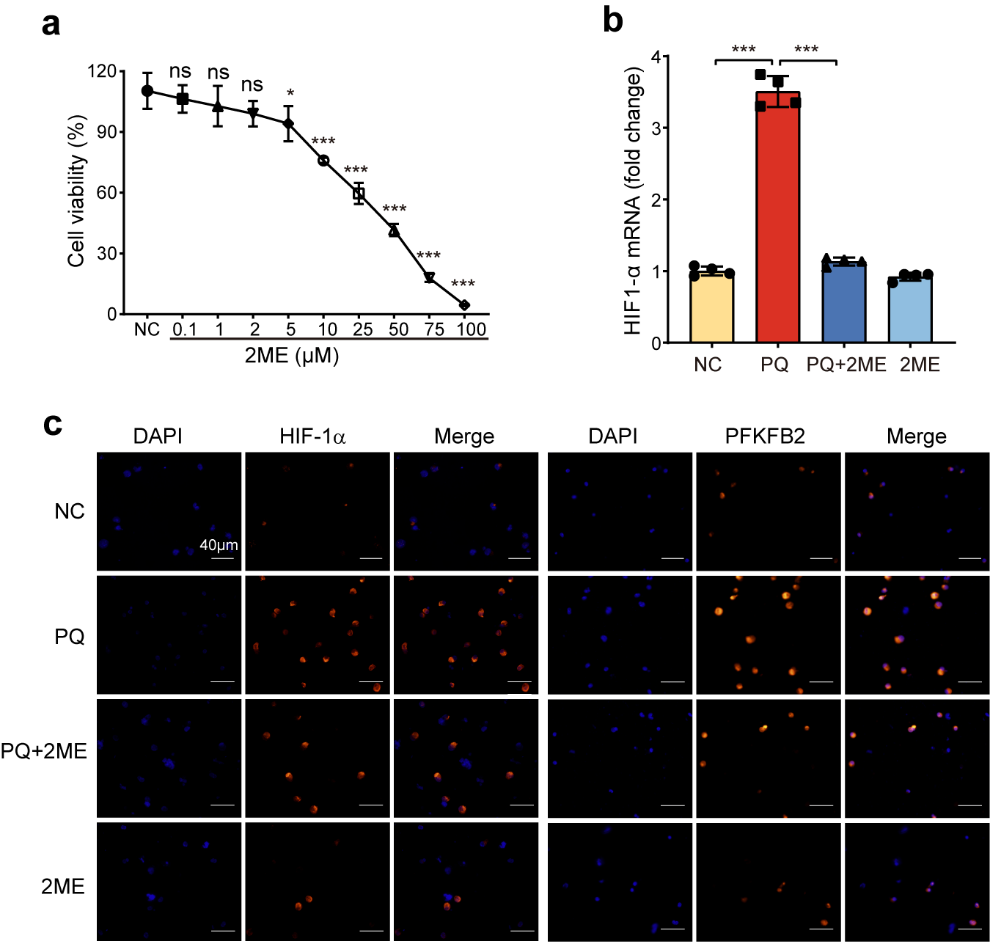


**Figure S6.** Cell toxicity analysis of 2ME and its effects on PFKFB2 and HIF-1α in BMDCs. a) Cell viability of BMDCs treated with various concentrations of 2ME (0, 0.1, 1, 2, 5, 10, 25, 50, 75, 100 μM) for 24h (n=4 wells/group). *^ns^P*>0.05, *^*^P*<0.05, *^**^P*<0.01, *^***^P*<0.001, compared to NC. b) qRT-PCR detection of PFKFB2 mRNA levels in BMDCs (n=4 wells/group). *^ns^P*>0.05, *^*^P*<0.05, *^**^P*<0.01, *^***^P*<0.001. c) Immunofluorescence detection of PFKFB2 and HIF-1α expression in BMDCs. Scale bar, 40 μm. All values are means ± SD, and significance was determined by one-way analysis of variance (ANOVA) with Fisher’s LSD post hoc analysis.

**
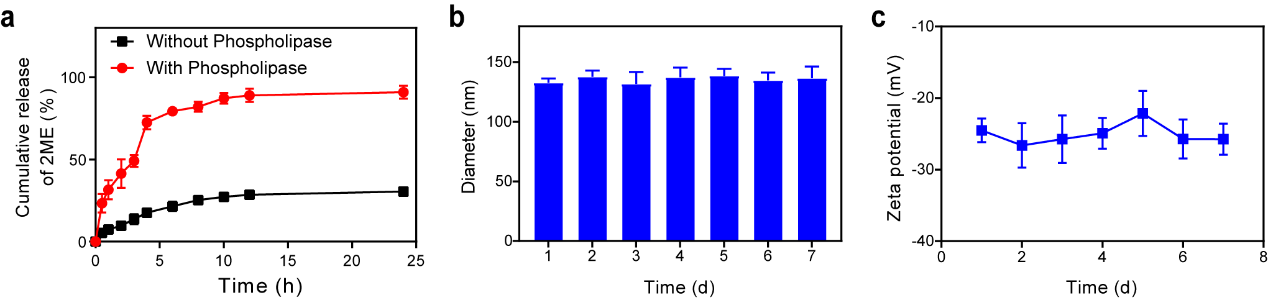
**

**Figure S7.** Characterization of 2ME NPs. a) Cumulative release of 2ME from DSPE-PEG-MAN nanoparticles measured over 24 h using the dialysis bag method in release medium with or without phospholipase. b,c) Changes in hydrodynamic diameter (measured daily via DLS) and zeta potential of 2ME-loaded DSPE-PEG-MAN nanoparticles during 4°C storage, showing <5% diameter variation and ±2 mV potential fluctuations respectively, indicating sustained colloidal stability. 2ME NPs: 2-methoxyestradiol@DSPE-PEG-MAN nanoparticles.

**
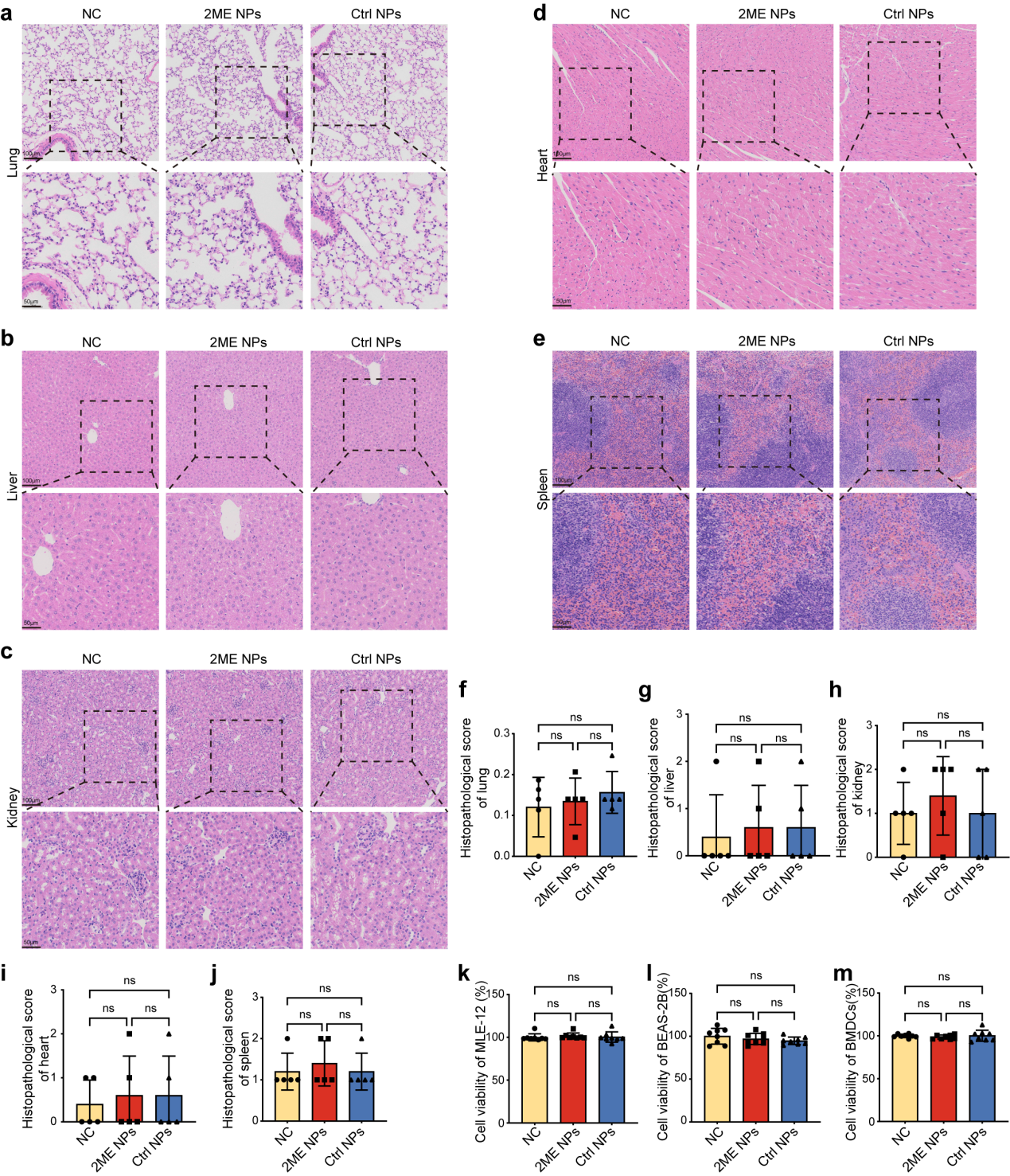
**

**Figure S8.** Safety assessment of 2ME NPs in vivo and in vitro. a-j) HE staining and pathological scoring of mouse tissues: lung, liver, kidney, heart, spleen (n=5 mice/gorup). k) MLE-12, l) BEAS-2B, and m) BMDCs cell viability after 24 h treatment with 2ME NPs or Ctrl NPs (2 μM) (n=8 wells/group for each cell line). *^ns^P*>0.05. All values are means ± SD, and significance was determined by one-way analysis of variance (ANOVA) with Fisher’s LSD post hoc analysis. 2ME NPs: 2-methoxyestradiol@DSPE-PEG-MAN nanoparticles, Ctrl NPs: Control nanoparticles (without 2ME loading).

**Table S1.** Comparison of hematological parameters and hepatic and renal function indicators in mice from different treatment groups.

| Parameters | NC | Ctrl NPs | 2ME NPs | *P* |
| --- | --- | --- | --- | --- |
| White Blood Cell Count (10^9/L) | 7.194±1.067 | 6.150±1.194 | 6.840±1.355 | 0.410 |
| Neutrophil Count (10^9/L) | 1.162±0.258 | 1.356±0.600 | 1.268±0.413 | 0.793 |
| Lymphocyte Count (10^9/L) | 5.376±1.001 | 4.622±1.626 | 4.088±1.488 | 0.373 |
| Monocyte Count (10^9/L) | 0.356±0.087 | 0.520±0.267 | 0.514±0.306 | 0.492 |
| Eosinophil Count (10^9/L) | 0.152±0.029 | 0.146±0.077 | 0.174±0.056 | 0.724 |
| Basophil Count (10^9/L) | 0.148±0.028 | 0.122±0.062 | 0.106±0.047 | 0.403 |
| Neutrophil Percentage (%) | 16.38±3.823 | 20.100±9.883 | 21.440±8.441 | 0.585 |
| Lymphocyte Percentage (%) | 74.42±4.170 | 66.580±15.610 | 65.100±14.020 | 0.463 |
| Monocyte Percentage (%) | 8.660±3.946 | 14.980±8.715 | 12.680±5.295 | 0.313 |
| Eosinophil Percentage (%) | 2.140±0.230 | 2.240±1.238 | 2.840±0.607 | 0.364 |
| Basophil Percentage (%) | 2.120±0.638 | 1.780±0.536 | 1.800±0.689 | 0.638 |
| Red Blood Cell Count (10^12/L) | 7.470±0.163 | 7.328±0.358 | 7.544±0.504 | 0.652 |
| Hemoglobin (g/L) | 124.200±3.271 | 121.600±2.702 | 123.600±9.182 | 0.767 |
| Hematocrit (%) | 40.060±0.832 | 39.400±1.355 | 39.520±2.483 | 0.811 |
| Mean Corpuscular Volume (fL) | 53.640±0.451 | 53.820±1.512 | 52.400±0.897 | 0.106 |
| Mean Corpuscular Hemoglobin (pg) | 16.620±0.164 | 16.620±0.661 | 16.380±0.444 | 0.657 |
| Mean Corpuscular Hemoglobin Concentration (g/L) | 309.400±5.459 | 309.000±6.892 | 312.600±5.128 | 0.584 |
| Red Cell Distribution Width - Coefficient of Variation (%) | 15.620±0.763 | 16.000±0.436 | 15.960±0.754 | 0.626 |
| Red Cell Distribution Width - Standard Deviation (fL) | 35.000±1.478 | 36.000±1.177 | 34.980±1.734 | 0.482 |
| Platelet Count (10^9/L) | 1023.000±150.900 | 960.600±214.100 | 1067.000±112.600 | 0.605 |
| Mean Platelet Volume (fL) | 6.120±0.259 | 6.280±0.045 | 6.320±0.311 | 0.393 |
| Platelet Distribution Width (fL) | 15.760±0.270 | 15.720±0.148 | 15.780±0.164 | 0.893 |
| Plateletcrit (%) | 0.623±0.072 | 0.603±0.137 | 0.671±0.069 | 0.547 |
| Uric Acid (U/L) | 155.200±32.190 | 153.400±10.930 | 176.100±13.940 | 0.212 |
| Urea Nitrogen (U/L) | 10.970±0.822 | 11.330±0.981 | 10.250±0.470 | 0.127 |
| Creatinine (mmol/L) | 22.230±8.557 | 17.200±9.221 | 21.170±9.915 | 0.672 |
| Aspartate Aminotransferase (U/L) | 100.200±19.210 | 115.100±16.430 | 109.800±18.680 | 0.445 |
| Alanine Aminotransferase (U/L) | 44.000±8.429 | 47.210±8.017 | 51.090±5.279 | 0.346 |
| Alkaline Phosphatase (mmol/L) | 106.700±12.360 | 108.400±16.840 | 98.590±14.980 | 0.552 |

All values are means ± SD, and significance was determined by one-way analysis of variance (ANOVA) . 2ME NPs: 2-methoxyestradiol@DSPE-PEG-MAN nanoparticles, Ctrl NPs: Control nanoparticles (without 2ME loading).

**
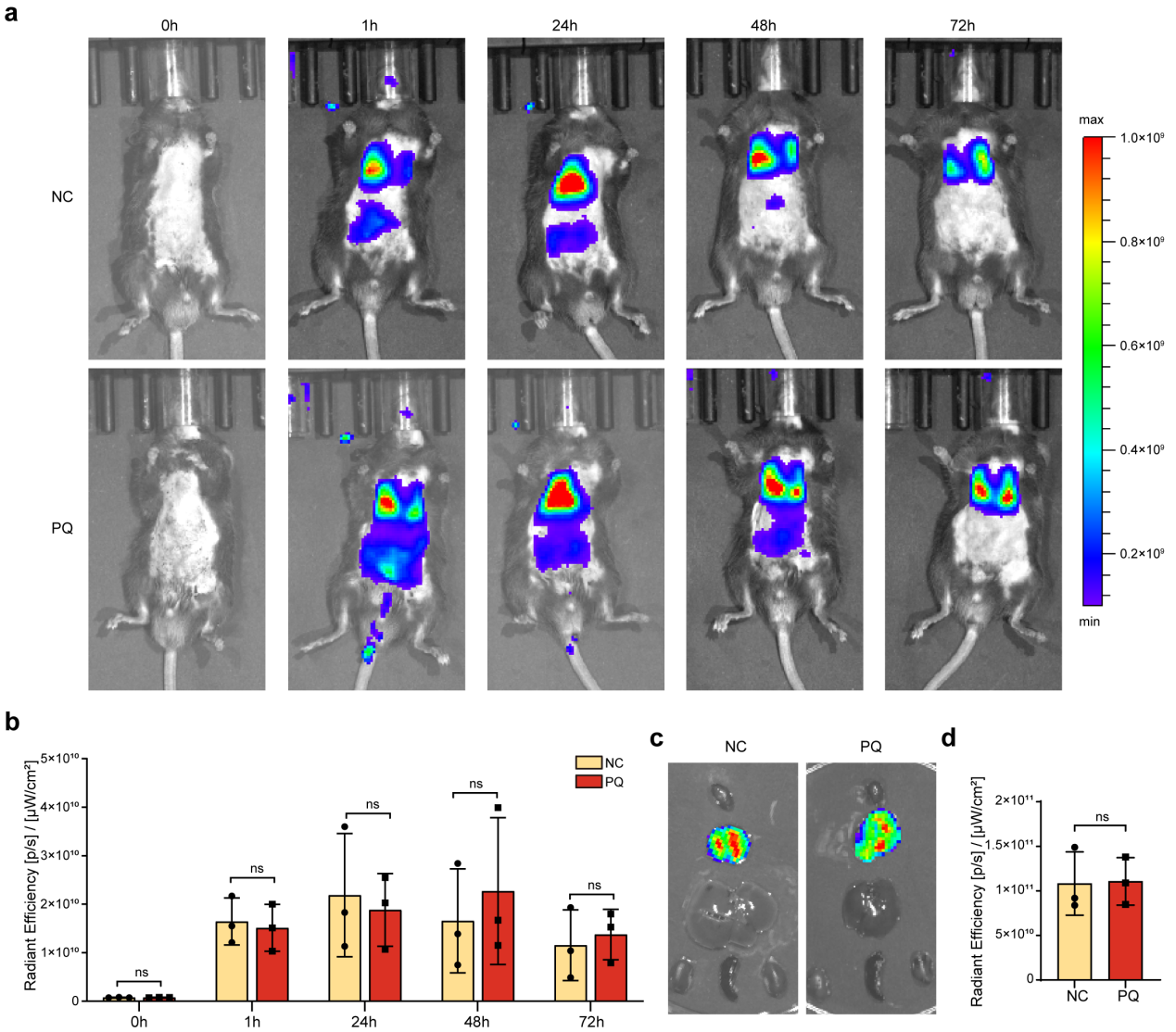
**

**Figure S****9.** Fluorescence imaging of mice after inhalation delivery of 2ME NPs. a,b) In vivo imaging of mice after inhalation of 2ME NPs-DIR and quantification (n=3 mice/group). c,d) Ex vivo imaging of major organs in mice after inhalation of 2ME NPs-DIR and quantification (n=3 mice/group). *^ns^P*>0.05, *^*^P*<0.05, *^**^P*<0.01, *^***^P*<0.001. All values are means  ± SD, and significance was determined by two-tailed Student’s t-test. 2ME NPs: 2-methoxyestradiol@DSPE-PEG-MAN nanoparticles.


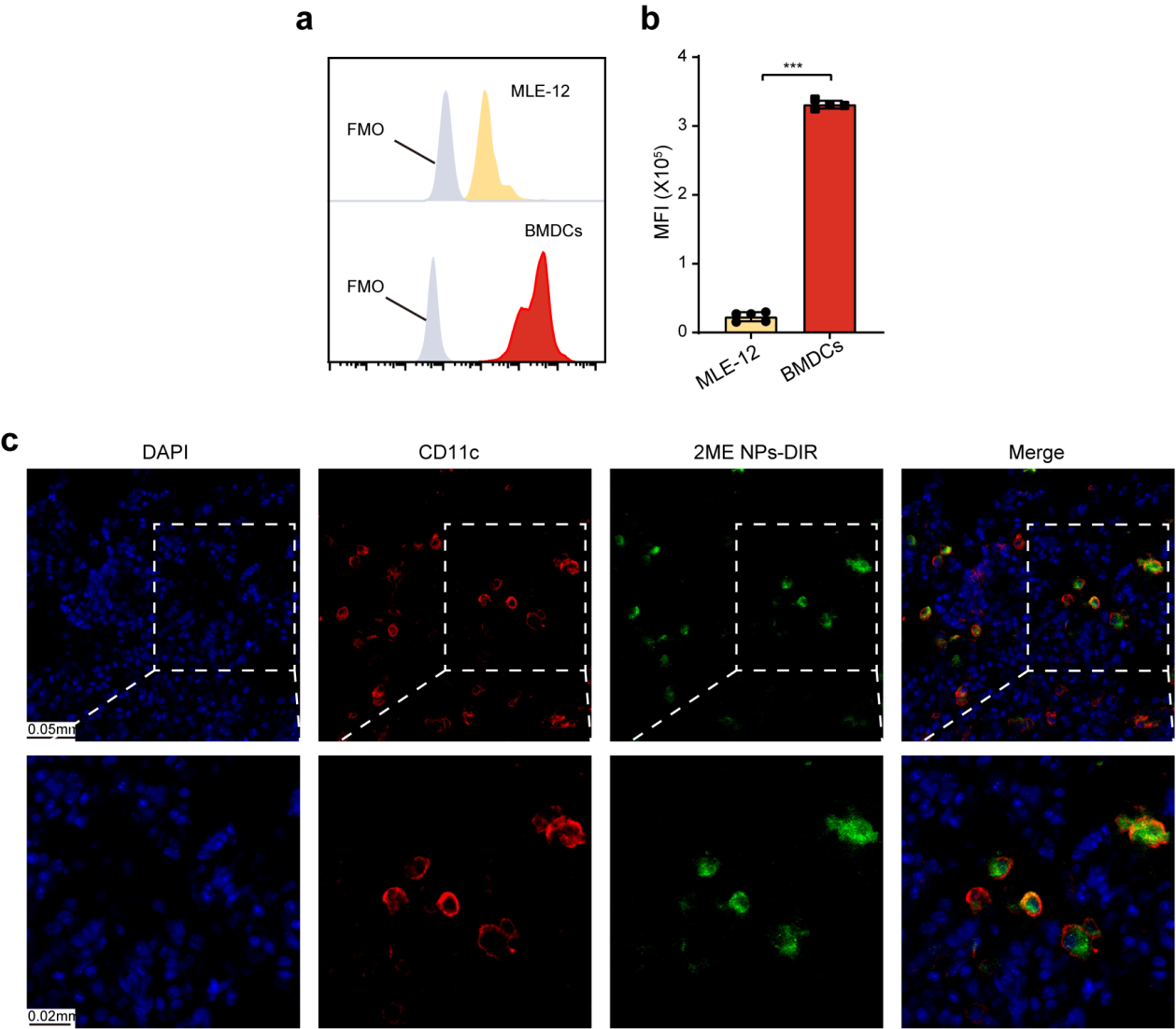


**Figure S10.** Analysis of the targeting of 2ME NPs to DCs. a,b) Flow cytometry analysis of fluorescence intensity in MLE-12 cells and BMDCs incubated with 2ME NPs-Cy5.5 (n=5 wells/group). c) Double immunofluorescence labeling with CD11c and 2ME NPs-DIR in the lungs of mice. *^ns^P*>0.05, *^*^P*<0.05, *^**^P*<0.01, *^***^P*<0.001. All values are means  ± SD, and significance was determined by two-tailed Student’s t-test. 2ME NPs: 2-methoxyestradiol@DSPE-PEG-MAN nanoparticles.


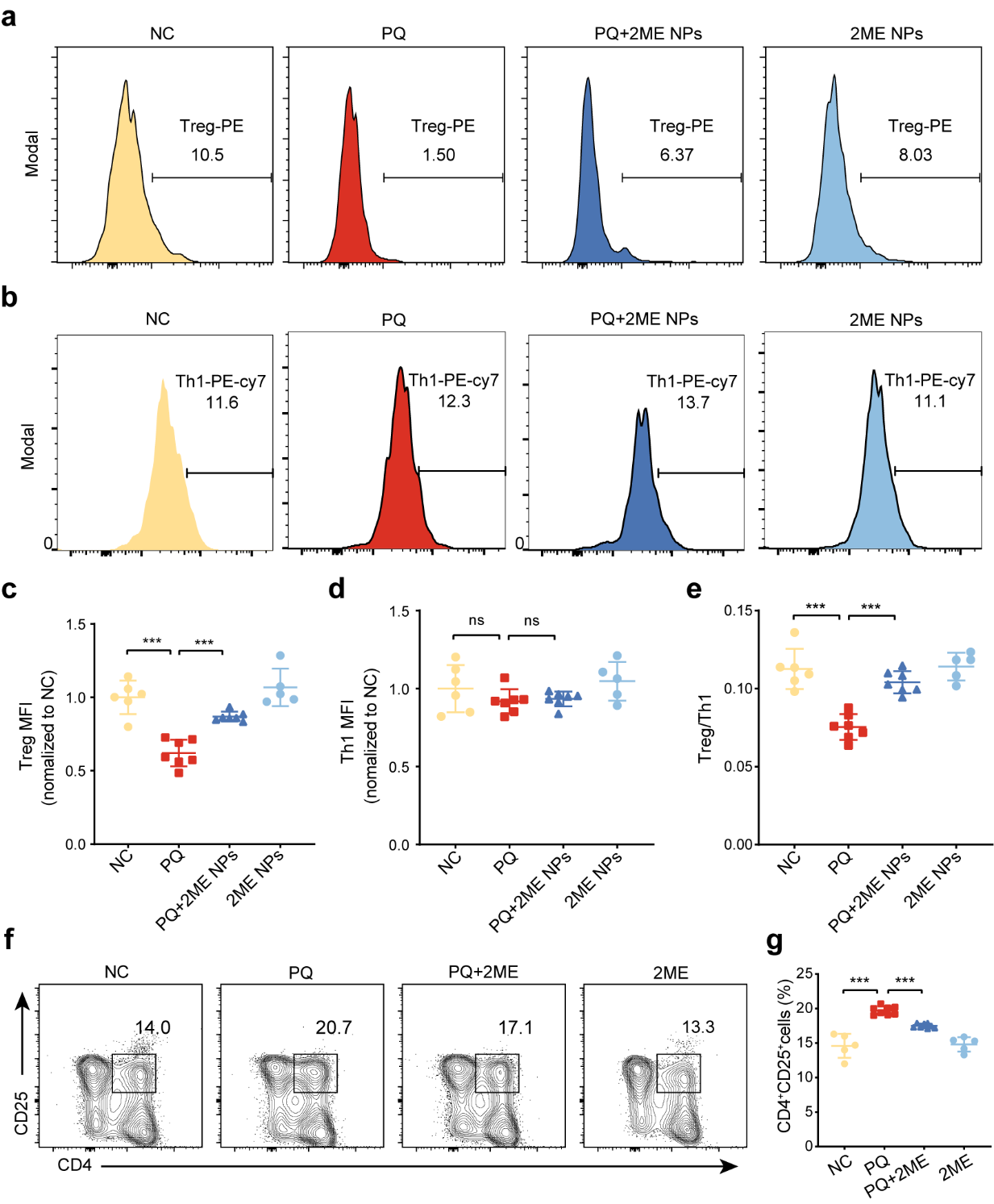


**Figure S11.** The role of HIF-1α in DCs-mediated T cell activation induced by PQ. a-e) Flow cytometry analysis of the proportions of Treg and Th1 cells in mouse lung tissue (n=5-7 mice/group). f,g) Flow cytometry analysis of the proportion of CD4⁺CD25⁺ subpopulation in T cells (n=5-7 samples/group). *^ns^P*>0.05, *^***^P*<0.001.All values are means ± SD, and significance was determined by one-way analysis of variance (ANOVA) with Fisher’s LSD post hoc analysis. 2ME NPs: 2-methoxyestradiol@DSPE-PEG-MAN nanoparticles.

**
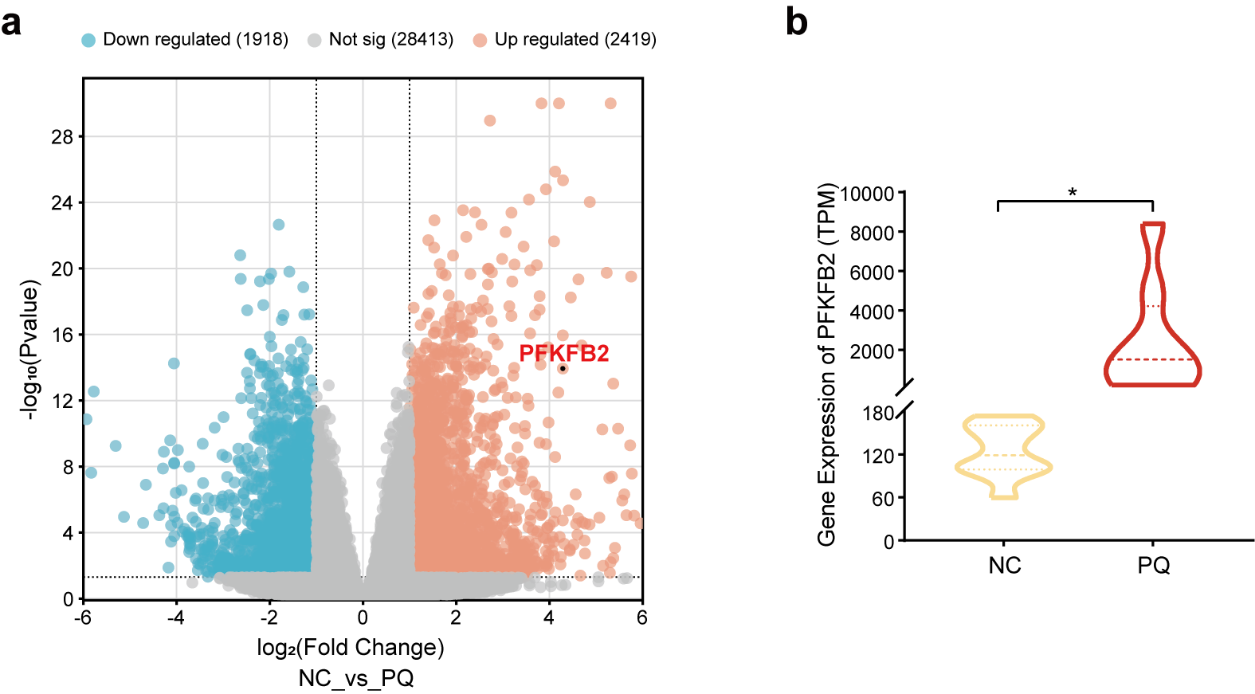
**

**Figure S12.** Peripheral blood transcriptomic analysis of PFKFB2 expression in PQ-exposed patients and normal controls (NC). a) Volcano plot showing differential gene expression between PQ-exposed patients (n=8) and NC (n=10). PFKFB2 is highlighted as one of the significantly upregulated genes in the PQ group. Genes with log₂(Fold Change) >1 and *P*<0.05 are marked in red (upregulated) or blue (downregulated), while non-significant genes are shown in gray. b) Quantitative analysis of PFKFB2 gene expression in peripheral blood. PFKFB2 expression is significantly elevated in PQ patients compared to NCs (n=8-10). *^*^P*<0.05. All values are presented as means ± SD, and significance was determined using a two-tailed Student’s t-test.

**Table S2.** General clinical data of patients with PQ poisoning and NC.

| Parameter | NC (n=10) | PQ (n=8) | *P* |
| --- | --- | --- | --- |
| Age (years) | 32.1 ± 14.13 | 30.0 ± 19.98 | 0.797 |
| Male, [cases (%)] | 8 (80) | 5 (62.5) | 0.410 |
| Blood PQ concentration  (μg/mL) | - | 0.42 (0.338, 0.8) | - |

Age was analyzed using the two-tailed Student’s t-test, and gender was analyzed using the Chi-square test.

**
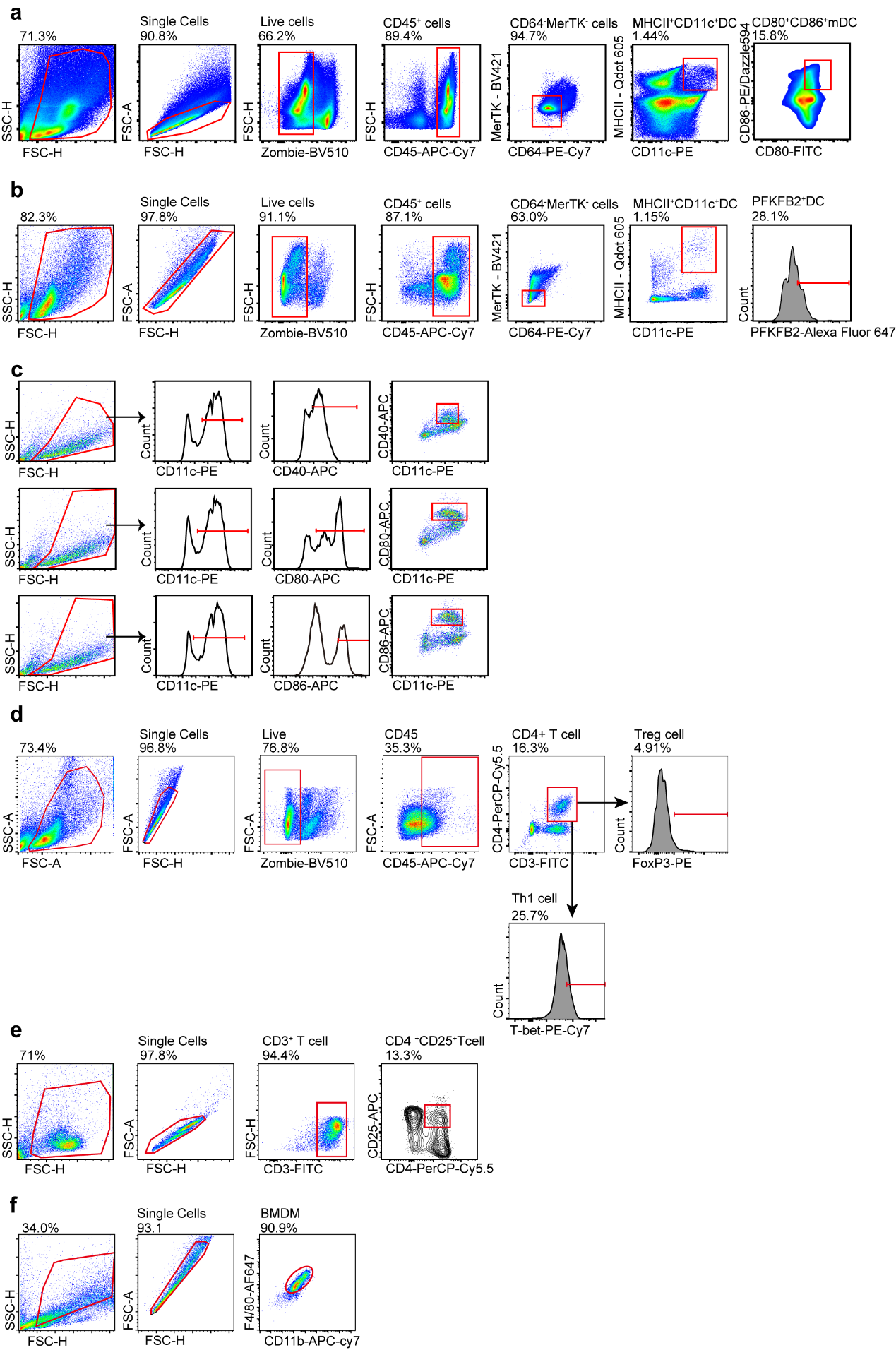
**

**Figure S13.** Gating strategies used for flow cytometry. a) Gating strategies for Figure 1b, 4i, 7i, 8i, 9i; b) Figure 7n, 9k; c) Figure 1e, 2h, 3i, 5e and S1f, S1h, S2b; d) Figure S11a, S11b; e) Figure S11f; f) Figure S4a.

| **Name** | **Application** | **Sequence (5’-3’)** |
| --- | --- | --- |
| PFKFB2 Probe 3’botin-1 | EMSA | GGGCCAAGCGCCCCACGTGACTCGCCCCAACCCCC |
| PFKFB2 Probe 3’botin-2 | EMSA | GGGGGTTGGGGCGAGTCACGTGGGGCGCTTGGCCC |
| Mut PFKFB2 Probe 3’botin-1 | EMSA | GGGCCAAGCGCCGGTGCACTCTCGCCCCAACCCCC |
| Mut PFKFB2 Probe 3’botin-2 | EMSA | GGGGGTTGGGGCGAGAGTGCACCGGCGCTTGGCCC |
| Cold-PFKFB2 Probe-1 | EMSA | GGGCCAAGCGCCCCACGTGACTCGCCCCAACCCCC |
| Cold-PFKFB2 Probe-2 | EMSA | GGGGGTTGGGGCGAGTCACGTGGGGCGCTTGGCCC |
| PFKFB2-F | ChIP | TGCAGGTGCTTCATTGGTCC |
| PFKFB2-R | ChIP | CACCCTATGACCTGTCCTATTCG |
| β-actin-F | qRT-PCR | GTGACGTTGACATCCGTAAAGA |
| β-actin-R | qRT-PCR | GTAACAGTCCGCCTAGAAGCAC |
| HIF-1α-F | qRT-PCR | GATGACGGCGACATGGTTTAC |
| HIF-1α-R | qRT-PCR | CTCACTGGGCCATTTCTGTGT |
| PFKFB2-F | qRT-PCR | ACATGCTCATGGGCTTCCTAT |
| PFKFB2-R | qRT-PCR | GTTGAGGTAGCGTGTTAGTTTCT |
| PFKFB2-F | Genotyping strategy | CTATCTTGCTAACCAGTCATCTTGC |
| PFKFB2-R | Genotyping strategy | CTAAACTACAGTGAGAGCTGGTAGG |
| CD11c-Cre-F | Genotyping strategy | ACTTGGCAGCTGTCTCCAAG |
| CD11c-Cre-R | Genotyping strategy | GCGAACATCTTCAGGTTCTG |

**Table S3.** The primer sequences for qRT-PCR, EMSA, ChIP and Genotyping strategy.
